# Altered Hypothalamic Protein Expression in a Rat Model of Huntington's Disease

**DOI:** 10.1371/journal.pone.0047240

**Published:** 2012-10-18

**Authors:** Wei-na Cong, Huan Cai, Rui Wang, Caitlin M. Daimon, Stuart Maudsley, Kerstin Raber, Fabio Canneva, Stephan von Hörsten, Bronwen Martin

**Affiliations:** 1 Metabolism Unit, National Institute on Aging, National Institutes of Health, Baltimore, Maryland, United States of America; 2 Receptor Pharmacology Unit, National Institute on Aging, National Institutes of Health, Baltimore, Maryland, United States of America; 3 Department for Experimental Therapy, Friedrich-Alexander-Universität Erlangen-Nürnberg, Erlangen, Germany; Federal University of Rio de Janeiro, Brazil

## Abstract

Huntington's disease (HD) is a neurodegenerative disorder, which is characterized by progressive motor impairment and cognitive alterations. Changes in energy metabolism, neuroendocrine function, body weight, euglycemia, appetite function, and circadian rhythm can also occur. It is likely that the locus of these alterations is the hypothalamus. We used the HD transgenic (tg) rat model bearing 51 CAG repeats, which exhibits similar HD symptomology as HD patients to investigate hypothalamic function. We conducted detailed hypothalamic proteome analyses and also measured circulating levels of various metabolic hormones and lipids in pre-symptomatic and symptomatic animals. Our results demonstrate that there are significant alterations in HD rat hypothalamic protein expression such as glial fibrillary acidic protein (GFAP), heat shock protein-70, the oxidative damage protein glutathione peroxidase (Gpx4), glycogen synthase1 (Gys1) and the lipid synthesis enzyme acylglycerol-3-phosphate O-acyltransferase 1 (Agpat1). In addition, there are significant alterations in various circulating metabolic hormones and lipids in pre-symptomatic animals including, insulin, leptin, triglycerides and HDL, before any motor or cognitive alterations are apparent. These early metabolic and lipid alterations are likely prodromal signs of hypothalamic dysfunction. Gaining a greater understanding of the hypothalamic and metabolic alterations that occur in HD, could lead to the development of novel therapeutics for early interventional treatment of HD.

## Introduction

Huntington's disease (HD) has a prevalence of 5–10 cases per 100,000 worldwide, which makes it the most common monogenetically inherited neurodegenerative disorder [1], [2]. HD is caused by expansion of a CAG repeat in the huntingtin (htt) gene [3]. The length of the CAG repeats correlates negatively with the age of onset and positively with severity of the disorder [4]. HD runs a debilitating and progressive course with an average survival of 10–25 years after disease onset [5]. Clinical presentations of HD typically include involuntary movements (especially chorea), progressive subcortical dementia [6], [7] and psychiatric disturbances [8]. Although the neurological symptoms predominate, they are not the sole pathophysiological manifestation of HD. Continuous reports, even before the discovery of the HD gene, described pathological phenotypes in peripheral tissues of HD patients, including weight loss [9]–[11], appetite hormone changes [12] and altered glucose homeostasis [13]–[15]. A growing body of evidence has also demonstrated other non-motor symptoms of HD that include impaired energy balance [16] and a disrupted circadian rhythm [17]. A recent study by Hult *et al.* [18] even established a causal link between mutant huntingtin expression in the hypothalamus and metabolic dysfunctions, and suggested that metabolic parameters are powerful readouts for assessing HD therapies.

The neuropathology of HD is characterized by intranuclear and cytoplasmic inclusions of huntingtin aggregates, and cell death primarily in the striatum and cerebral cortex. Striatal atrophy [19] is well established in clinical diagnosis of HD and has been linked to chorea, whereas changes in the cerebral cortex have been correlated with cognitive decline [20]–[23]. In light of non-motor symptoms and signs of perturbed functionality of the hypothalamus in HD, it has been hypothesized that this region may also be affected in HD. Supporting this hypothesis, Soneson *et al.* found distinguishable changes in the hypothalamic region of HD gene carriers by MRI [24]. Consistently, hypothalamic atrophy was also observed in transgenic HD mice using voxel-based morphometry (VBM) of MRI images [25]. Changes in neuropeptide levels can also indicate hypothalamic dysfunctions. In cross-sectional cerebrospinal fluid (CSF) samples collected from HD patients, Bjorkqvist *et al.* reported significantly increased levels of Cocaine- and amphetamine-regulated transcript (CART) peptide. This neuropeptide has strong anorectic effects [26]. In line with this, Gabery *et al.* quantified the neuropeptide-expressing hypothalamic neurons known to regulate metabolism and confirmed the increase of CART-expressing neurons in HD patients [27]. Taken together, the evidence suggests that the delicate balance of the neuroendocrine system, which mainly depends on the hypothalamus, could be altered and likely contributes to the peripheral tissue abnormalities frequently observed in HD. However, the proteomic alterations underlying HD-related hypothalamic changes are still unclear.

With the discovery of the htt mutation in 1993, it became possible to create animal models with a similar genetic background to the disease phenotype seen in humans with HD. The R6/2 mouse is the most commonly used transgenic mouse model of HD. R6/2 mice develop symptoms as early as 4 weeks, although the average age at onset of symptoms is 9 to 11 weeks. The mean lifespan for this model is 10 to 13 weeks, and animals rarely survive past 14 weeks [28]. The N171-82Q transgenic mouse is another model for HD. Beginning at week 11, these mice show a progressive decline in performance on the rotarod test as well as the onset of clasping behavior [29]. The lifespan of N171-82Q mice is no more than 20 weeks. Early onset of symptoms and short lifespan of both these animal models restrain the depth of possible HD research. In order to overcome these problems, the only transgenic rat model currently in use for HD was created by von Hörsten and colleagues [30]. These rats express 51 human-derived CAG repeats inserted in a 1962 bp rat htt fragment of complementary DNA (cDNA). This relatively small number of CAG repeats results in an adult-onset progressive phenotype. In this transgenic rat model, motor symptoms appear at 10 to 15 months of age and the average lifespan can reach 24 months [31]. Due to the late onset of symptoms and extended lifespan compared to mouse models of HD, the tgHD rat model allows for more comprehensive evaluations such as those required to investigate metabolic and neuroendocrine function in a longitudinal manner.

As a “body-wide” disorder, symptomatic progression of HD is tightly linked with peripheral metabolic health [32]. In order to better understand the pathogenesis of hypothalamic dysfunctions in HD, we carried out a comprehensive longitudinal metabolic characterization of the tgHD rat model in the current study. Furthermore, we used the tgHD rat model to delineate alterations occurring in the hypothalamus at the proteomic level.

## Results

### Alterations in circulating euglycemic hormone levels in pre-symptomatic and symptomatic tgHD rats

**Figure 1:**
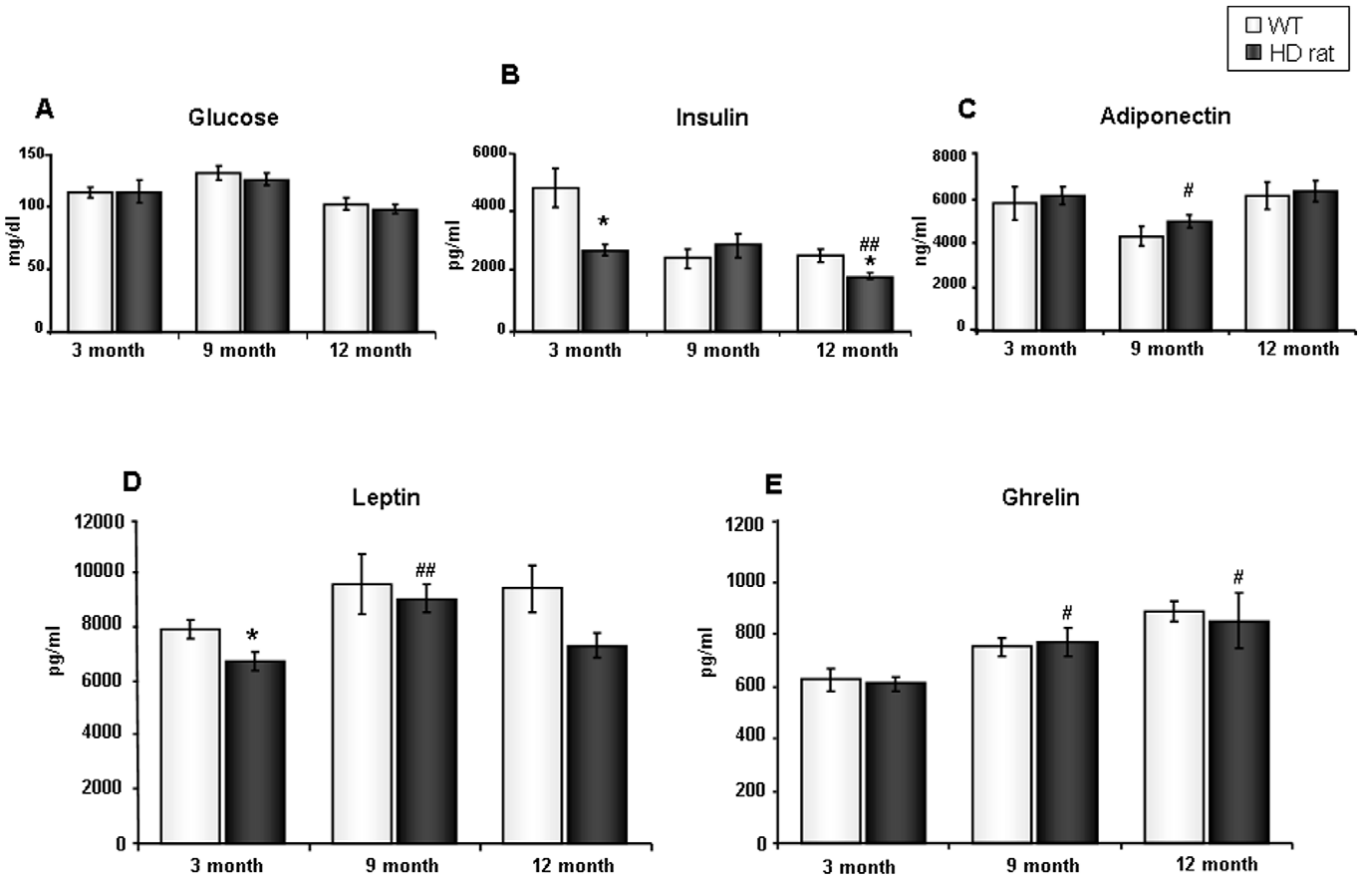
Altered plasma glycemic hormone and appetite hormone levels in tgHD rats. (A) Mean blood glucose concentrations for WT and tgHD rats across three ages. (B) Mean plasma insulin concentrations in WT and tgHD rats across three ages. (C) Mean plasma adiponectin concentrations in WT and tgHD rats across three ages. (D) Mean plasma leptin concentrations in WT and tgHD rats across three ages. (E) Mean plasma total ghrelin concentrations in WT and tgHD rats across three ages. Values are mean ± SEM, *: p<0.05; tgHD vs. WT, #: p<0.05; ##: p<0.01; 9-month or 12 month tgHD vs. 3-month tgHD, n = 5 per group.

We assessed longitudinal circulating levels of glucose, insulin, adiponectin, ghrelin (total), and leptin in rats aged 3-months (pre-symptomatic), 9-months (early-symptomatic) and 12 months (symptomatic). Longitudinal analysis demonstrated that tgHD rats possessed comparable levels of circulating glucose, compared to the WT rats across all three ages (Fig. 1A). However, significantly lower insulin levels were detected in both 3-month and 12-month tgHD rats, compared to the WT controls (p = 0.013, 3-month tgHD vs. 3-month WT; p = 0.028, 12-month tgHD vs. 12-month WT, Fig. 1B). Longitudinally, both tgHDs and WTs exhibited a progressive reduction in plasma insulin levels (p = 0.0069, 12-month tgHD vs. 3-month tgHD; p = 0.009, 9-month WT vs. 3-month WT; p = 0.008, 12-month WT vs. 3-month WT). There was no significant difference in plasma adiponectin levels between tgHDs and WTs across all three ages. When compared to 3-month tgHD animals, the 9-month tgHD rats displayed a significant decrease in adiponectin levels (Fig. 1C). In order to better understand peripheral metabolic alterations in tgHD rats, we further evaluated levels of leptin and ghrelin, two major appetite-modulating hormones. Compared to WT animals, the 3-month old tgHD rats demonstrated a significant decrease in plasma leptin levels (p = 0.044, Fig. 1D). There was also a trend towards reduction in the 12-month tgHD rats (p = 0.067), as well. Interestingly, compared to the 3-month tgHD animals, 9-month tgHD rats revealed a significant increase in leptin levels (p = 0.0067, Fig. 1D). Longitudinally, both tgHD rats and WT rats displayed a progressive elevation in plasma ghrelin levels (p = 0.028, 9-month tgHD vs. 3-month tgHD; p = 0.043, 12-month tgHD vs. 3month tgHD; p = 0.002, 12-month WT vs. 3-month WT) (Fig. 1E). The euglycemic hormone alterations demonstrated in the tgHD rats are similar to what has been shown previously in mouse models of HD (N171-82Q, [15]). Additionally, we also performed two-way ANOVA analysis. Two-way ANOVA analysis allows us to simultaneously consider the effects from two independent factors, *i.e.* genotype and age. With 2-way ANOVA analysis, as shown in Table S1, similar results were obtained: significantly lower insulin levels were detected in 3-month tgHD rats, compared with age-matched WTs. Compared to 12-month WT animals, the 12-month tgHD rats demonstrated a significant decrease in plasma leptin levels. Longitudinally, 9 month tgHD rats demonstrated significantly higher leptin levels compared to 3-month tgHD animals. A significant age-dependent increase in ghrelin was also found in both tgHD and WT rats.

### Longitudinal alterations in metabolic hormones in the tgHD rat

**Figure 2:**
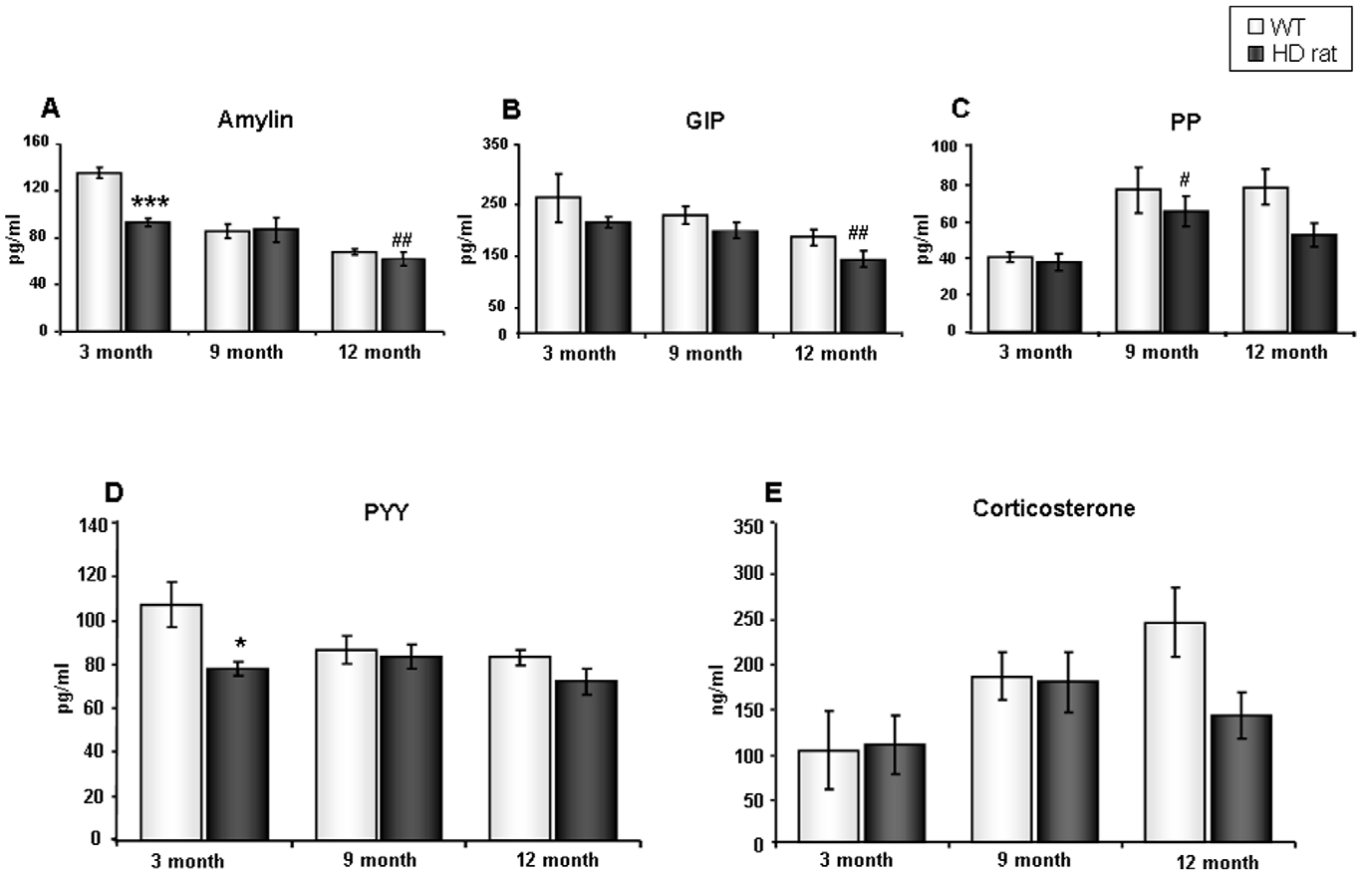
Alterations in metabolic hormones in tgHD rats. (A) Mean plasma amylin concentrations for WT and tgHD rats across three ages. (B) Mean plasma GIP concentrations in WT and tgHD rats across three ages. (C) Mean plasma PP concentrations in WT and tgHD rats across three ages. (D) Mean plasma PYY concentrations in WT and tgHD rats across three ages. (E) Mean plasma corticosterone concentrations in WT and tgHD rats across three ages. Values are mean ± SEM, *: p<0.05; ***: p<0.001; tgHD vs. WT, #: p<0.05; ##: p<0.01; 9-month or 12 month tgHD vs. 3-month tgHD, n = 5 per group.

We measured circulating levels of numerous metabolic hormones including amylin, gastric inhibitory polypeptide (GIP), pancreatic peptide (PP), and Peptide YY (PYY). We found that amylin (Fig. 2A) and PYY (Fig. 2D) levels were significantly decreased in 3-month tgHD rats compared to WT controls (p = 0.0001 for amylin; p = 0.025 for PYY). Compared to 12-month WT rats, we observed decreasing trends in both GIP (p = 0.072) (Fig. 2B) and PP (p = 0.078) (Fig. 2C) in 12-month tgHD rats. In addition, both tgHD and WT animals displayed significant age-dependent reductions in amylin levels (p = 0.002, 12-month tgHD vs. 3-month tgHD; p<0.001, 9-month WT vs. 3-month WT; p<0.001, 12-month WT vs. 3-month WT, Fig. 2A). Compared to 3-month tgHD rats, 12-month tgHD rats exhibited significantly decreased GIP levels (Fig. 2B), whereas 9-month tgHD rats demonstrated a significant increase in PP levels (Fig. 2C). WT rats also showed profound age-related elevations in their PP levels (p = 0.02, 9-month WT vs. 3-month WT; p = 0.006, 12-month WT vs. 3-month WT, Fig. 2C). In addition to evaluating metabolic hormone levels, we also evaluated levels of corticosterone, a stress-responsive hormone. We found that only 12-month tgHD rats demonstrated a trend of reduction in systemic corticosterone (p = 0.068, vs. 12-month WT) (Fig. 2E). Additionally, we also used 2-way ANOVA to assess potential changes in plasma amylin, GIP, PP, PYY and corticosterone levels. Consistently, we observed a significant decease in amylin and PYY levels in 3-month tgHD rats, compared to 3-month WT rats (Table S1).

### Alterations in circulating lipid levels in the tgHD rat

**Figure 3:**
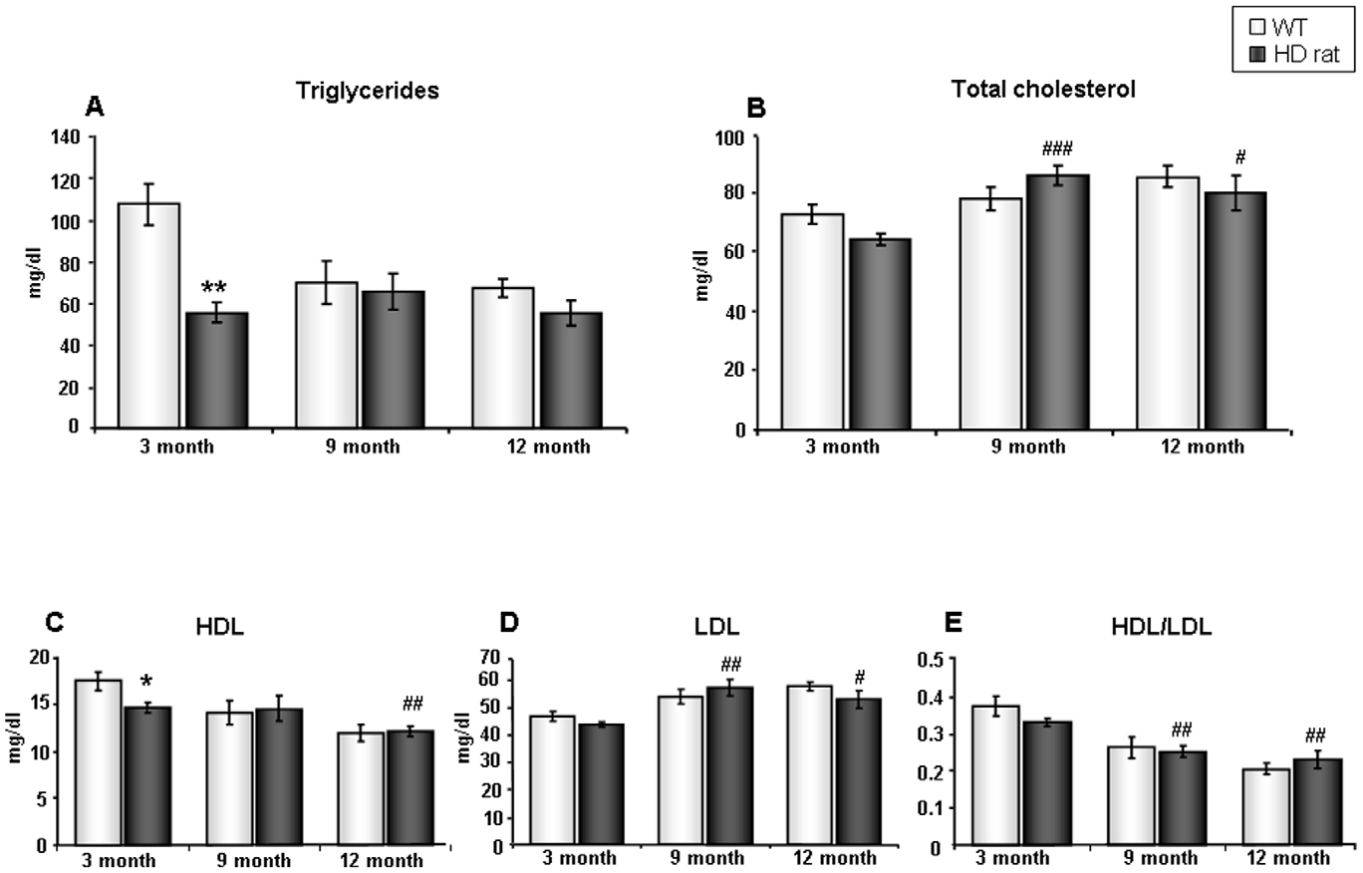
Changes in plasma lipid levels in tgHD rats. (A) Mean plasma triglyceride levels for WT and tgHD rats across three ages. (B) Mean plasma total cholesterol levels in WT and tgHD rats across three ages. (C) Mean plasma HDL levels in WT and tgHD rats across three ages. (D) Mean plasma LDL levels in WT and tgHD rats across three ages. (E) Mean ratio of HDL/LDL in WT and tgHD rats across three ages. Values are mean ± SEM, * : p<0.05; * * : p<0.01; tgHD vs. WT, #: p<0.05; ##: p<0.01; ###: p<0.001; 9-month or 12 month tgHD vs. 3-month tgHD, n = 5 per group.

We next analyzed plasma lipid levels including triglycerides, total cholesterol, high density lipoprotein (HDL), and low-density lipoprotein (LDL). The triglyceride levels of 3-month tgHD rats were significantly reduced compared to WT animals (p = 0.0025, vs. 3-month WT). However, this difference was abolished in both 9-month and 12-month tgHD rats. In addition, WT rats, but not tgHD rats, exhibited age-related significant decreases in plasma triglycerides (p = 0.046, 9-month WT vs. 3-month WT; p = 0.008, 12-month WT vs. 3month WT, Fig. 3A). The tgHD rats and WT rats shared similar total cholesterol levels, except for the 3-month tgHD rats, which showed a trend of reduction (p = 0.056, vs. 3-month WT). Longitudinally, compared to 3-month tgHD rats, both 9-month and 12-month tgHD rats possessed markedly increased levels of total cholesterol (p = 0.0005, 9-month tgHD vs. 3-month tgHD; p = 0.0378, 12-month tgHD vs. 3month tgHD, Fig. 3B). For plasma lipoproteins, a significant decrease was detected in HDL of 3-month tgHD rats compared to age-matched WTs (p = 0.032, Fig. 3C). No statistical difference between tgHD and WT animals was observed with respect to LDL levels (Fig. 3D). Similarly, there was no difference between tgHD and WT animals with respect to the ratio of HDL to LDL (Fig. 3E). Both 12-month tgHD and WT rats had lower HDL levels than their 3-month counterparts. Longitudinally, in terms of LDL and HDL/LDL, both tgHD rats and WT rats showed similar age-dependent changes (Fig. 3E). Using 2-way ANOVA analysis, again, we obtained similar plasma lipid results (Table S1). Triglyceride levels in 3-month old tgHD rats were significantly reduced compared to the age-matched WT animals. Longitudinally, compared to the 3-month old tgHD rats, both 9-month and 12-month tgHD rats possessed markedly increased levels of total cholesterol. Additionally, 3-month old tgHD rats also demonstrated markedly lower LDL levels, compared to 9-month or 12-month tgHD rats. In terms of the ratio of HDL/LDL, tgHD rats demonstrated an obvious age-dependent reduction.

### Proteomic alterations in the hypothalamus of tgHD rats

**Figure 4:**
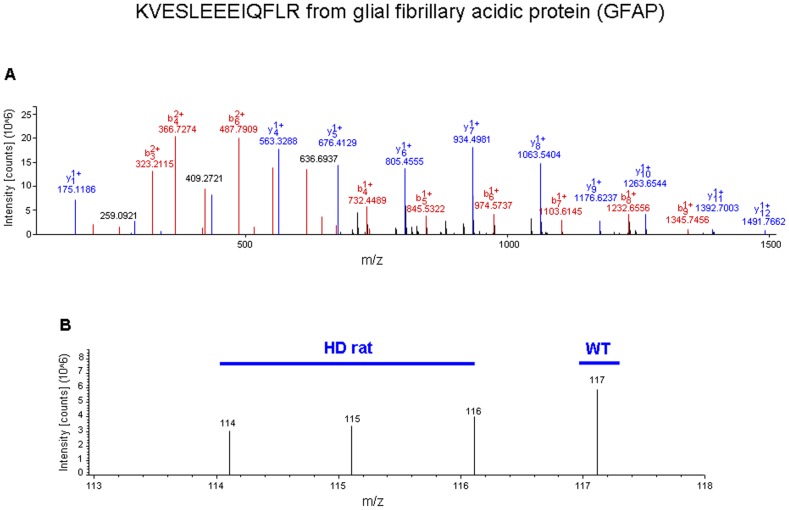
Sample iTRAQ analysis and iTRAQ ratio (tgHD: WT) for glial fibrillary acidic protein (GFAP) in hypothalamus. (A) Full scan MS/MS event for a single identified parent ion from GFAP with its multiple b and y series daughter ions is shown. (B) Successively expanded MS/MS spectra for identifying the low mass range end of the MS/MS scan event. The isobaric mass tag labels added were 114, 115 and 116 for tgHD rat samples and 117 for WT control samples.

**Figure 5:**
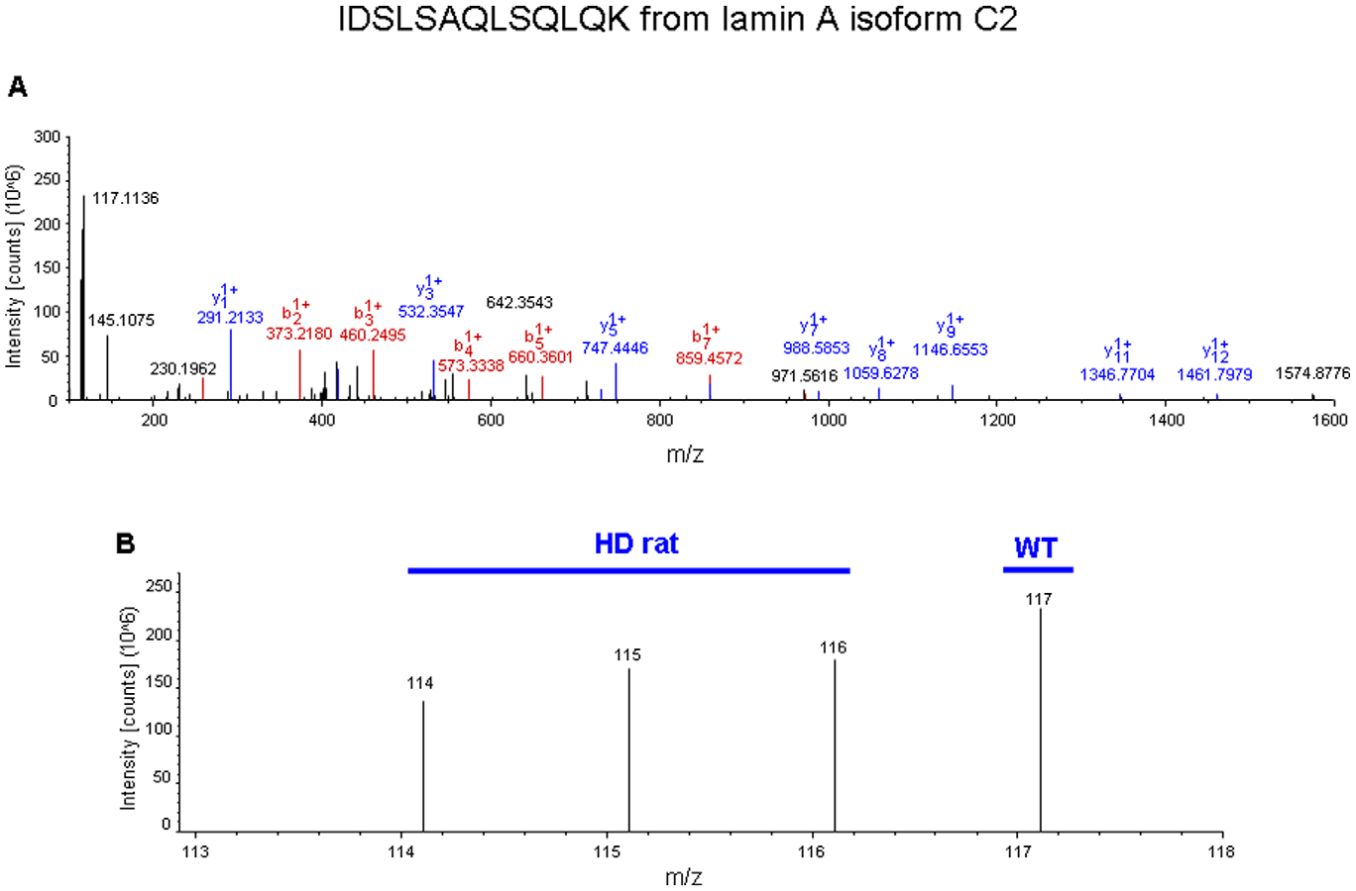
Sample iTRAQ analysis and iTRAQ ratio (tgHD: WT) for lamin A isoform C2 (Lamin A) in hypothalamus. (A) Full scan MS/MS event for a single identified parent ion from Lamin A with its multiple b and y series daughter ions shown. (B) Successively expanded MS/MS spectra for identifying the low mass range end of the MS/MS scan event. The isobaric mass tag labels added were 114, 115 and 116 for tgHD rats samples and 117 for WT control samples.

**Figure 6:**
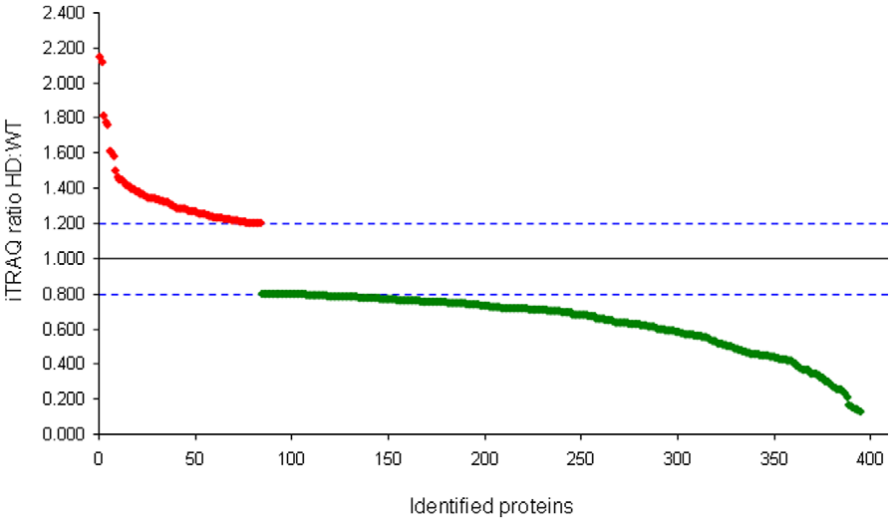
iTRAQ ratios of tgHD rat versus WT control for hypothalamus. Mean tgHD (114, 115,116) to the WT (117) isobaric mass tag ratios for samples resolved by MS/MS from the hypothalamus of both WT and tgHD rats. Proteins identified from peptides that displayed a ratio greater than or equal to 1.2 or less that or equal to 0.8 are considered to be statistically different according to standard protocols from unity and therefore distinctly regulated compared to WT.

**Figure 7:**
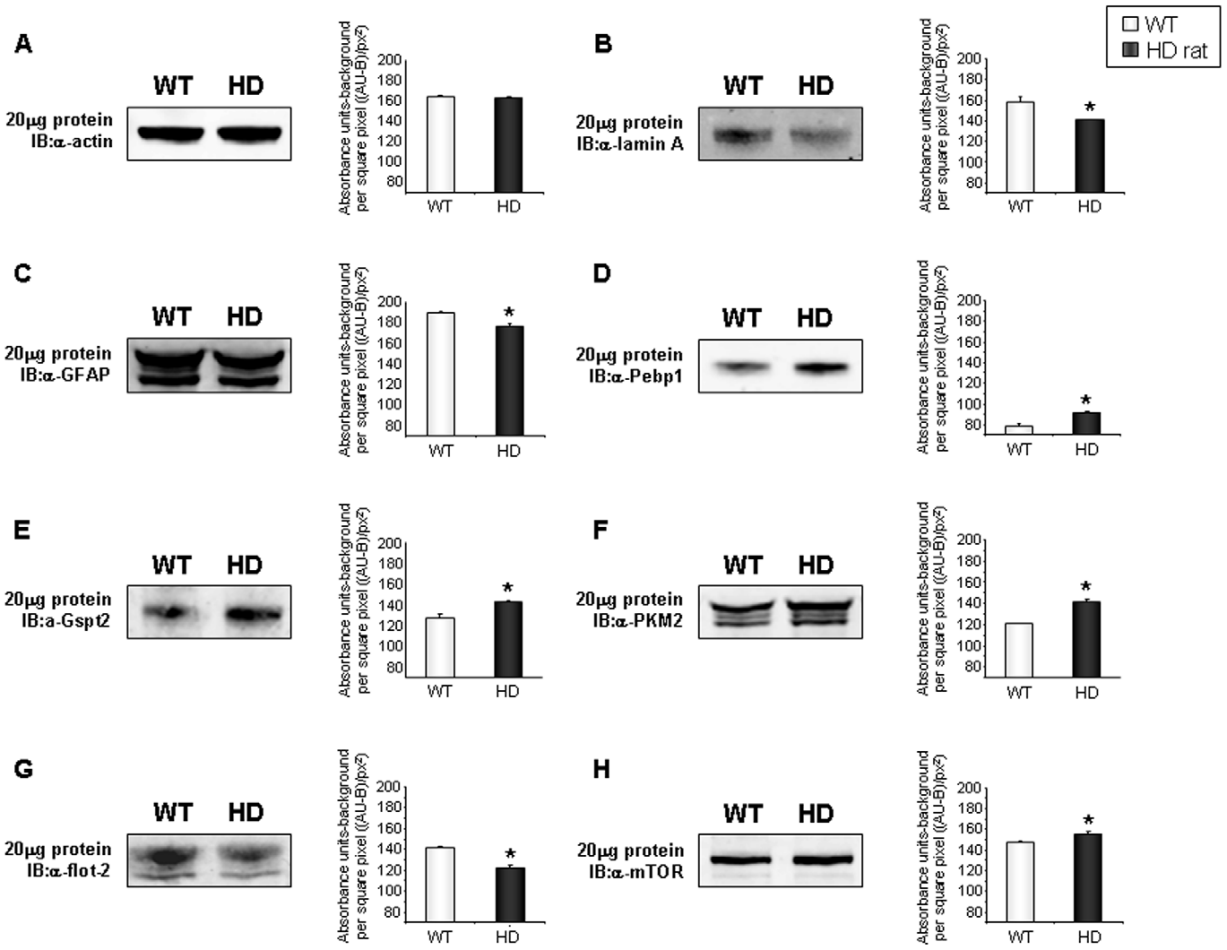
Western blot validation of multiple significantly regulated proteins identified by iTRAQ in tgHD hypothalami. Quantification of the representative western blots is indicated for the WT (white bars) and HD (dark bars) for the respective proteins: Lamin A (B); GFAP (C); Pebp1 (D); Gspt2 (E); PKM2 (F); Flot-2 (g); and mTOR (H). Expression variation was normalized to actin (A) expression. Assessment of statistical significance was performed with GraphPad Prism (version 5) using a Student's t-test. * : p≤0.05. Values are mean ± SEM, n = 5 per group.

iTRAQ mass tag ratios were calculated for proteins with reliable identifications (see Materials and Methods) based upon their collision-induced dissociation fragmentation patterns. Ratios of the tgHD mass tags (114, 115, 116) compared to the WT tags (117) that were greater than 1.2 or less than 0.8 were considered to be statistically different, *i.e.* altered expression level of the protein. Two representative iTRAQ mass spectrometry peptide identifications (glial fibrillary acidic protein (GFAP) and Lamin A) are demonstrated in Figs. 4 and 5. In total, we identified 395 significantly altered proteins in the hypothalamus of the tgHD rats (compared to the WT), which included 84 up-regulated proteins and 311 down-regulated proteins (Fig. 6, Table S2). To verify the expression changes of the identified proteins, western blots were performed by resolving tissue lysate samples onto PVDF membranes (Fig. 7). Seven proteins that were identified as significantly altered proteins by iTRAQ mass spectrometry were selected for western blot analysis. Among these proteins, G1 to S phase transition 2 (Gspt2), phosphatidylethanolamine binding protein 1 (Pebp1), muscle pyruvate kinase (Pkm2) and mTOR were up-regulated in the hypothalamus and GFAP, flotillin-2 (Flot2), and Lamin A were down-regulated in the hypothalamus of tgHD rats. Westen blotting of these proteins revealed differential protein expression, consistent with the iTRAQ results.

### Phenotypic physiological signaling pathway analysis of protein alterations in tgHD rats

**Figure 8:**
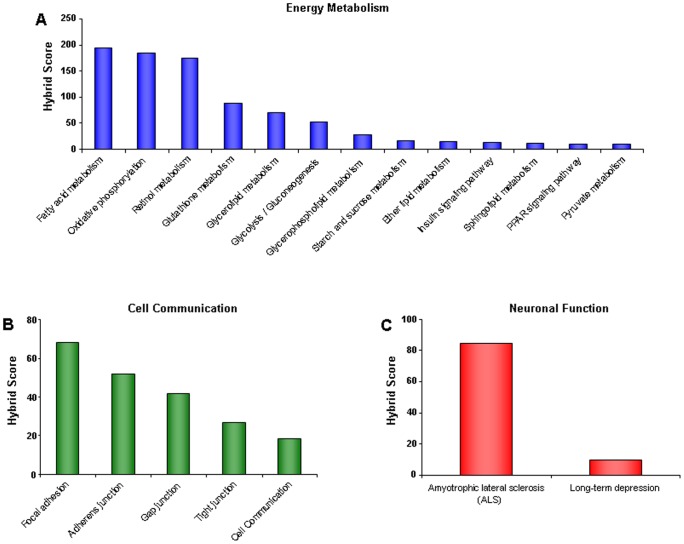
KEGG signaling pathway analysis of significantly regulated hypothalamic proteins. Proteins significantly regulated in tgHD rats compared to WT controls were used as input data for KEGG signaling pathway population analysis. The significantly populated pathways for tgHD rats were rationally clustered into subgroups focused upon energy metabolism (A), cell communication (B), and neuronal function (C). For each significantly-populated KEGG pathway a ‘hybrid’ score was generated which represents the −log_10_ of the enrichment probability multiplied with the relative enrichment factor compared to the background proteomic expression.

**Figure 9:**
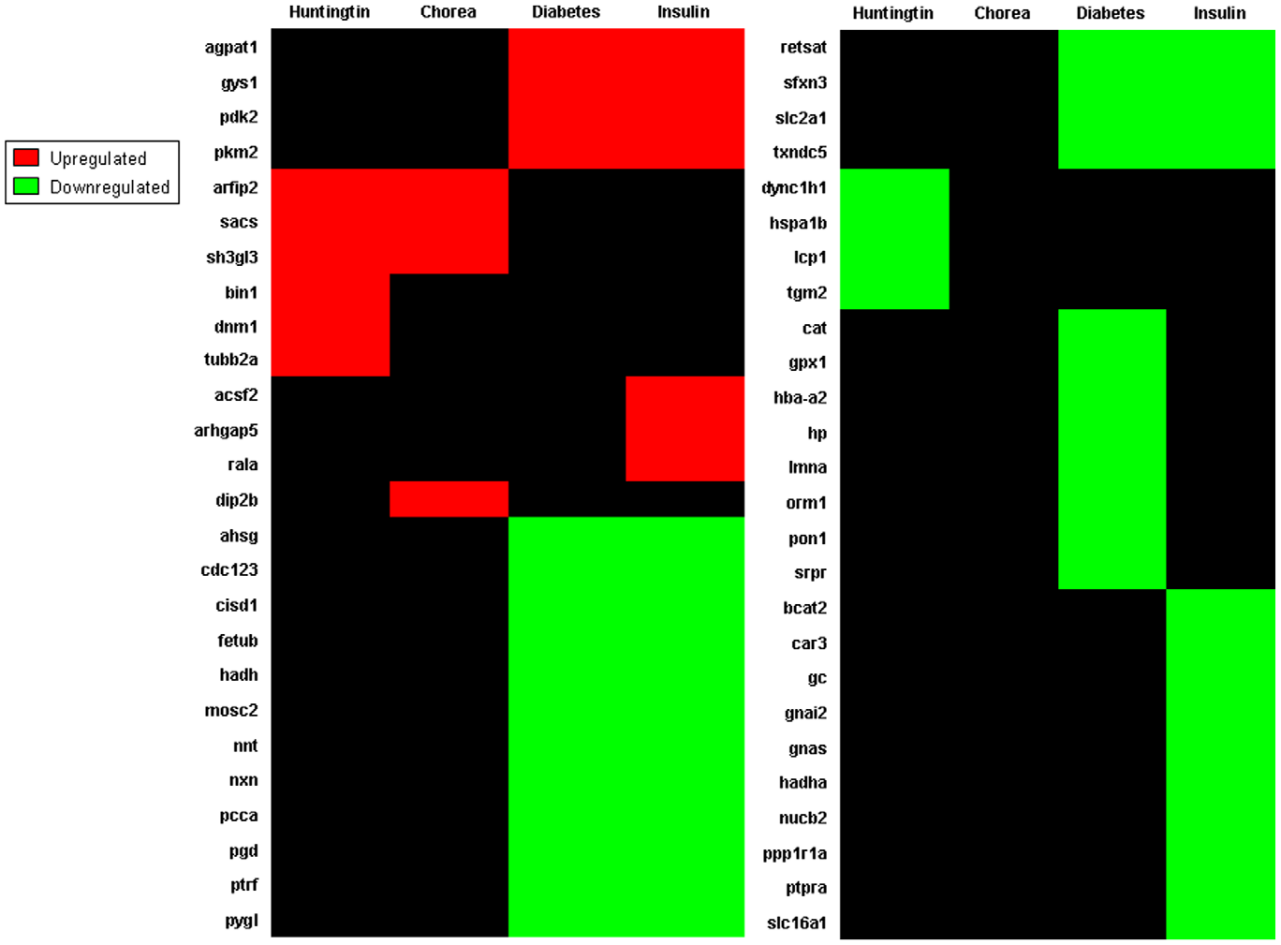
LSI correlation analysis of altered hypothalamic proteins in TgHD rats. LSI analysis of tgHD significantly-regulated hypothalamic proteins was performed using the following search terms: Huntingtin, Chorea, Diabetes and Insulin. Red signifies up-regulated proteins and green signifies down-regulated proteins.

Both up- and down-regulated protein sets identified by iTRAQ were analyzed using the KEGG pathway algorithm. Use of the KEGG pathway algorithm allows for the potential physiological role(s) of significantly altered proteins to be further delineated. We found that a major body of significantly altered proteins in tgHD rats was closely associated with energy metabolism. Calculation of subsequent hybrid scores further revealed that the significantly altered proteins were highly involved in *fatty acid metabolism*, *oxidative phosphorylation*, *glycolysis/gluconeogenesis*, *glycerolipid metabolism*, *PPAR signaling pathway* and the *insulin signaling pathway* (Fig. 8, Table 1). Other significantly altered proteins were strongly associated with cell communication (*Focal adhesion*, *Adherens junction*, *Gap junction*, *Tight junction*) and neuronal function (*Amytrophic Lateral Sclerosis*, *Long-term depression*, Fig. 8, Table 1). To further appreciate the potential hypothalamic pathophysiological ramifications of these protein alterations, we performed LSI analysis on the significantly altered proteins (Fig. 9). LSI analysis revealed that many proteins were significantly correlated to Huntingtin (arfaptin-2 (Arfip2), sacsin (Sacs), heat shock 70 kD protein 1B (Hspa1b), transglutaminase 2 (Tgm2)), chorea (Arfip2, Sacs, disco-interacting protein 2 homolog B (Dip2b)), diabetes (1-acylglycerol-3-phosphate O-acyltransferase 1 (Agpat), glycogen synthase 1 (Gys1), solute carrier family 2(Slc2a1)), and insulin (pyruvate dehydrogenase kinase isozyme 2 (Pdk2), pyruvate kinase 2 (Pkm2), branched chain aminotransferase 2 (Bcat2), carbonic anhydrase 3 (Car3)). In line with the results obtained from the KEGG analysis, upon application of LSI analysis we found that the strongest proteomic representation for tgHD rats was significantly correlated to the energy-related terms ‘diabetes’ and ‘insulin’. A full list of LSI analysis results is provided in Table S3 (up-regulated) and Table S4 (down-regulated).

**Table 1:** KEGG group clustering of significantly altered hypothalamic proteins in tgHD rats compared to wild-type controls.

Up-regulated proteins	Hybrid Score
Glutathione metabolism	93.9
Fatty acid metabolism	93.9
Gap junction	80.0
Starch and sucrose metabolism	62.0
Glycerophospholipid metabolism	53.6
Insulin signaling pathway	16.9
Purine metabolism	16.5
Focal adhesion	10.2

Down-regulated proteins	Hybrid Score
Fatty acid elongation in mitochondria	269.4
Oxidative phosphorylation	257.9
Complement and coagulation cascades	238.3
Retinol metabolism	234.0
Valine, leucine and isoleucine degradation	218.8
Benzoate degradation via hydroxylation	185.3
Fatty acid metabolism	123.0
Amyotrophic lateral sclerosis (ALS)	115.9
Propanoate metabolism	107.3
Bile acid biosynthesis	85.9
Leukocyte transendothelial migration	78.7
Adherens junction	73.8
Regulation of actin cytoskeleton	72.7
Focal adhesion	62.2
Glycerolipid metabolism	57.6
Calcium signaling pathway	53.2
Glycolysis/Gluconeogenesis	44.2
Metabolism of xenobiotics by cytochrome P450	41.8
Tight junction	39.0
Glutathione metabolism	37.8
Nitrogen metabolism	34.9
beta-Alanine metabolism	32.7
Limonene and pinene degradation	30.7
Antigen processing and presentation	28.6
Cell Communication	26.8
Arginine and proline metabolism	23.8
Cell adhesion molecules (CAMs)	19.8
Nicotinate and nicotinamide metabolism	15.5
Tryptophan metabolism	15.4
Long-term depression	13.8
ECM-receptor interaction	11.9
Butanoate metabolism	11.9
Gap junction	11.3
Lysine degradation	11.2
Fructose and mannose metabolism	10.4
Glycerophospholipid metabolism	7.8
Taste transduction	7.4
Axon guidance	5.8
Insulin signaling pathway	5.2

KEGG group clusters were only extracted from the differentially-regulated protein list if at least two proteins were present in each group with a compound probability of p≥0.05. To account for both KEGG enrichment (R: Observed expression frequency/Expected expression frequency) and compound probability (p) of clustered proteins, a hybrid score was used. Hybrid score = R * −log_10_(p).

## Discussion

Currently, as multiple lines of evidence have demonstrated widespread pathophysiologies in peripheral systems of both HD patients and model organisms [33], it is clear that HD is more than a purely neurological motor disorder [34]. In this study, we demonstrated significant proteomic alterations in the hypothalamus of tgHD rats, and this was accompanied by significant alterations in circulating levels of metabolic hormones and lipid factors. Unbiased bioinformatics analyses (KEGG and LSI), revealed that many pathways and proteins associated with diabetes and insulin signaling were significantly altered in the tgHD rats. Longitudinal assessments of glycemic hormones at 3-months, 9-months and 12-months of age demonstrated that even in pre-symptomatic animals (3 months) there were significant alterations in glycemic and metabolic hormones (Fig. 1, 2). In the pre-symptomatic stage, tgHD rats demonstrated significant decreases in levels of insulin, leptin, and two gut hormones (amylin and PYY). These findings are consistent with the fact that some non-motor symptoms of HD precede the onset of motor symptoms by several years in patients [35] as well as by several months in the rat model [31]. In our present study, we applied both Student's t-test analysis and 2-way ANOVA analysis for the statistical evaluation of all plasma parameters. The Student's t-test allowed us to perform comparisons between tgHD rats and WT rats of each age. In the longitudinal analysis, the Student's t-test was used to compare 3-month animals with the other two ages within same genotype group. In addition to this, we also performed a 2-way ANOVA analysis which allowed us to compare two independent variables *i.e.* genotype and age at the same time.

In the clinical setting, HD patients typically develop diabetes mellitus approximately seven times more often than matched control participants [36]. Recent evidence has also demonstrated that abnormal glucose homeostasis in HD patients can be, in-part, attributed to impaired insulin secretion capacity [37]. In addition to the characteristic neurological symptoms, weight loss is also a prominent feature of HD, suggesting that considerable metabolic abnormalities are present in this disorder. Results from one clinical study [35] have subsequently shown that altered circulating leptin and ghrelin levels are strongly linked with the dysregulation of bodyweight in HD patients. In line with this clinical evidence, dysregulation of insulin and leptin in the pre-symptomatic stage tgHD rats could also be an early sign of poor glycemic control and altered appetite, which typically occurs in mid to late stages of HD [38], [15]. Amylin, or Islet Amyloid Polypeptide (IAPP), is a 37-residue peptide hormone secreted by pancreatic β-cells at the same time as insulin (in a roughly 1∶100 amylin∶insulin ratio). Peptide YY (PYY), is found in the endocrine cells of the duodenum and exocrine pancreas, and inhibits gastric emptying and secretin-stimulated pancreatic secretion. The changes in amylin and PYY observed in the tgHD rats could further suggest impaired insulin secretion [39], [40] or lack of insulin-like function, which could result in additional abnormalities in glucose homeostasis in HD. Clinically, IAPP has been shown to be co-secreted from the pancreas into the circulation with insulin in response to glucose- or amino acid-triggered insulin secretion. Hyposecretion of IAPP and insulin [41] is highly characteristic of a Type 1 diabetic state, which shares many functional and physiological aspects with the dysglycemic condition present in both HD patients [42] and rodent HD models [43].

Interestingly, in the early symptomatic animals (9-months), the difference between tgHD and WT rats in insulin, leptin, amylin and PYY were no longer apparent. It is likely that at this stage of the disease, compensatory mechanisms were activated in order to try and regain homeostatic balance [44]. However, in the symptomatic stage (12-months), alterations in metabolic hormones were apparent, including decreased GIP and PP levels. GIP is a duodenal peptide named for its ability to inhibit gastric acid secretion, but its most potent effects include its ability to mediate insulin release and its strong insulinotropic effects on adipose tissue and liver. The potent insulinotropic action of GIP, while predominantly on the pancreatic islet beta-cells, may also involve an intraislet regulation of alpha cells [45]. Pancreatic polypeptide (PP) is expressed only in the pancreatic islets. PP inhibits pancreatic secretion of enzymes, bicarbonate, and water, and may play a role in the negative feedback control of the pancreas through its inhibitory action on vagal acetylcholine release [46]. The alterations in GIP and PP potentially indicate abnormalities related to pancreatic secretion, which may affect both insulin and glucagon metabolism [47], [48]. In support of this, we have previously shown that there are significant alterations in pancreatic islet of Langerhans morphology and distribution of insulin and glucagon secreting cells in N171-82Q HD mice [15]. Recent evidence has also shown that GIP (glucose-dependent insulinotropic peptide) analogues can elicit beneficial effects upon synaptic plasticity and neurogenesis [49]. It is plausible therefore that a decrease of circulating GIP could be one of the hallmarks for the neurodegenerative processes that occur in HD. Even with such knowledge it is clear that further clinical HD patient studies are needed to help uncover the specific causative links between metabolic hormones such as GIP and PP and the pathophysiological etiology of HD.

Another crucial functional component of energy metabolism, plasma lipids, were also investigated in this tgHD rat model. Again, even in the pre-symptomatic stage, the tgHD rats showed a statistically significant reduction in both triglycerides and HDL. Alterations in triglycerides in tgHD rats could be due to adipose tissue dysfunction, which has been demonstrated in two HD mouse models, the R6/2 mice and CAG140K1 mice [50]. Consistently, the reduction of plasma and CSF leptin levels present in HD patients also suggests a disruption of adipose tissue function in this neurological disorder [35]. A growing body of evidence suggests that whole brain cholesterol metabolism is impaired in both HD patients [51] and HD animal models [52]. However, in our study, we did not detect any significant differences between tgHD rat and WT animals with respect to total circulating cholesterol and LDL levels. It is possible that the circulating cholesterol pool may buffer the alterations in the brain cholesterol pool. Interestingly, we also observed a profound decrease in HDL in the 3-month tgHD rats. A similar finding was reported in a recent clinical study in which HD patients demonstrated dramatically decreased HDL levels, when they displayed altered growth hormone response in the arginine test [53].

A growing body of research has established a strong association between HD and hypothalamic dysfunction such as appetite changes [16], [54], circadian rhythm disturbance [55], and gut hormone alterations [15]. These hypothalamic defects can further lead to the unintended loss of bodyweight and dysfunctional energy metabolism such as diabetes and generalized insulin resistance. However, the associated underlying hypothalamic alterations are still unclear. To address this, we applied an iTRAQ proteomic approach to elucidate molecular alterations that may occur during the clinical course of HD. In the present study, we used isoflurane inhalation as an acute anesthesia method, which allows us to complete hypothalamus collection in less than 8 minutes. We specifically expedited our hypothalamic tissue collection, and minimized excessive isoflurane exposure in this as well as our previous study [56], to attenuate any potential effects our extraction procedure may have had upon protein expression or post-translational modification status. Thus, we could investigate, in a quantitative manner, proteomic changes in the hypothalamus. We identified a total of 395 significantly regulated proteins, which included 84 up-regulated proteins and 311 down-regulated proteins. As expected, the majority of the top-regulated proteins are closely related to neurophysiological functions, such as glial fibrillary acidic protein (Gfap), lamin A, annexin A2 (Anxa2) and Ras-related protein Ral-A precursor (Rala). GFAP is the main intermediate filament protein in mature astrocytes, but is also an important component of the cytoskeleton in astrocytes during development. It has been shown that GFAP is involved in astrocyte functions, which are important during regeneration, synaptic plasticity and reactive gliosis [57]. The altered Lamin A/C, is associated with changes in nuclear morphology (*e.g.* increased nuclear volume). Homozygous defects in LaminA cause autosomal recessive axonal neuropathy in human (Charcot-Marie-Tooth Disorder Type 2) and mouse [58]. Anxa2 heterotetramer is involved in learning and neuronal activities [59]. RalA depletion or ectopic expression of constitutively active RalA in cultured neurons inhibits axon formation [60]. Some altered proteins are even more specifically linked to pathology of Huntington's disease, such as Heat shock protein-70 (Hspa1b), cytoplasmic dynein 1 heavy chain 1 (Dync1h1). Hsp70 is a molecular chaperone which colocalizes to inclusion bodies, which are neuroprotective in HD animal models. Recent research has also demonstrated that Hsp70 functionally interacts with soluble mutant huntingtin oligomers in a classic ATP-dependent manner [61]. Dynein dysfunction is a potential causal mechanism in neurodegenerative diseases, most notably in degenerative diseases of the striatum such as Huntington's disease [62]. In addition, we also discovered profound alterations in several oxidative stress-responsive proteins, such phospholipid hydroperoxide glutathione peroxidase (Gpx4) [63], glutathione S-transferase Mu 2 (Gstm2) and glutathione S-transferase Mu 7 (Gstm7) [64]. In order to better understand the changes of those proteins, we also used unbiased clustering of proteins into functional pathways (KEGG) as this allows for a physiological ‘phenotype’ of the protein set to be created that is not entirely dependent on specific protein identities in the set [65]. Using KEGG pathway analysis, the major pathways that were found to be altered in the tgHD rat hypothalami included: *fatty acid metabolism*, *oxidative phosphorylation*, *glycerolipid metabolism*, *glycolysis/gluconeogenesis*, *insulin signaling pathway*, and *PPAR signaling pathway*. In addition to KEGG analysis, LSI analysis revealed that the significantly altered proteins were highly associated with the input interrogation terms ‘diabetes’ and ‘insulin’. Implicitly-correlating proteins of note discovered using this informatic process included glycogen synthase1 (Gys1), pyruvate dehydrogenase kinase2 (Pdk2), pyruvate kinase M2 (Pkm2), 1-acylglycerol-3-phosphate O-acyltransferase 1 (Agpat1) and acyl-CoA synthetase family member 2 (Acsf2). Gys1, the rate-limiting enzyme in the synthesis of glycogen, is glycogen synthase encoded by two genes: Gys1 is expressed in muscle and other tissues, and Gys2, primarily expressed in liver (regulating liver glycogen synthase). Since conversion to glycogen is a major fate of ingested glucose in the body, reducing glycogen synthase activity can lead to severe glucose intolerance [66]. Pdk2 is a member of a family of pyruvate dehydrogenase kinases (Pdks 1–4). Four dedicated Pdk isoenzymes have been identified, each of which display a distinct tissue-specific expression profile, and have differential regulatory properties. Pdk plays a key role in controlling the balance between glucose and lipid oxidation according to substrate supply [67]. Increasing glucose oxidation by inhibiting Pdk may be an effective mechanism to increase glucose utilization; additionally, increasing pyruvate oxidation may further contribute to lowering glucose levels by decreasing the supply of gluconeogenic substrates [68]. The glycolytic key regulator Pkm2 (or M2-PK) can switch between a highly active tetrameric and an inactive dimeric form. In its active form, M2-Pk/Pkm2 is a metabolic sensor, which regulates cell proliferation, cell growth, and apoptotic cell death in a glucose supply-dependent manner [69]. Taken together, these three proteins play critical roles in the process of glycolysis/gluconeogenesis. The fact that we observed significant alterations in these proteins in the tgHD rat hypothalamus suggests that there is possible involvement of dysfunctional glycolysis/gluconeogenesis in HD. Most cells synthesize their glycerophospholipids and triglycerides (TG) to maintain cellular integrity and to provide energy for cellular functions. Agpat1 controls serial acylations of glycerol-3-phosphate, which is involved in *de novo* phospholipid synthesis in cells [70]. Acsf2 is one of enzymes that mediates fatty acid transportation and catalyzes the formation of acyl-CoA from fatty acids, ATP, and CoA [71]. Interestingly, these two lipid synthesis enzymes were dramatically up-regulated in the hypothalamus of the tgHD rat, which suggests increased activity of lipid synthesis in the hypothalamus. As signals of nutrient abundance, alterations of hypothalamic lipid levels might further contribute to changes in feeding behavior and other peripheral metabolic dysfunctions in HD [72]. Although it would also be interesting to investigate distinct proteomic profiles in microregions of the hypothalamus, this is not currently possible with iTRAQ technology, due to the large protein amounts that are required (150 µg).

In this study, we have gained a greater appreciation of the intricacies of alterations in the neuroendocrine system occurring throughout the Huntington's disease process. In assessing the presence of pathophysiology, one must realize that the tissue or organ that is diseased is not merely a passive entity and that its cells may react in a specific manner to the imposed insult [73]. This theory has been partly testified in our longitudinal results of tgHD rat model. Therefore, in certain important organ(s) or tissue(s) such as the hypothalamus, complicated signaling pathways and functional groups of proteins may reveal that both positive and negative regulatory mechanisms are working in concert to create the resultant diseased phenotype. By using proteomic techniques, such as iTRAQ, we gained a deeper understanding of the complex signaling pathways and functional groups of proteins that are altered in the hypothalamus of HD. With these findings, we can better understand and interpret the powerful metabolic readouts from peripheral tissues of HD and potentially open up novel therapeutic avenues for the treatment of HD.

## Materials and Methods

### Animals, blood collection and tissue harvesting

Male transgenic HD (tgHD) rats, containing a truncated huntingtin cDNA fragment with 51 CAG repeats under the control of the naïve rat huntingtin promoter and their male wild type (WT) littermates were used for this study. All rats were kept under a 12∶12 h light-dark cycle with the light period starting at 6:00 am. All animals received *ad libitum* access to food and water. All animal procedures were approved by the Animal Care and Use Committee of the Friedrich-Alexander-Universität Erlangen-Nürnberg, Germany and conducted according the international guidelines for the use and care of laboratory animals. Longitudinal analyses were conducted on male tgHD rats and their age- and gender-mated WT littermates. Blood samples were collected from the tail vein from each animal (n = 5 per group) after an overnight fast at the following ages: 3-months (pre-symptomatic), 9-months (early symptomatic), and 12-months (symptomatic). At 12 months of age, animals were euthanized using inhalation isoflurane (∼1 ml/430 cm^3^) and decapitation. Hypothalami were carefully dissected out and were stored at −80°C for proteomic analyses.

### Measurement of circulating metabolic hormones and lipids

Plasma glucose levels were measured using an enzymatic assay kit (GO Assay, Sigma, St. Louis, MO), according to the manufacturer's instructions. Plasma metabolic hormones were measured using a rodent multiplex assay kit (Millipore, Billerica, MA), as described previously [74], [75]. Each sample was assayed in duplicate on a 96-well plate. Analysis of quality control standards provided in the kit met expectations, validating the accuracy of panel. Additionally, this assay had an inter-assay precision of <23% and an intra-assay precision of <7%. Plasma lipid levels including triglycerides, total cholesterol, high density lipoprotein (HDL), and low density lipoprotein (LDL) were assayed using enzymatic methods with commercial kits (Wako Chemicals USA, Inc., Richmond, VA), according to the manufacturer's instructions. Plasma corticosterone levels were measured using standard radioimmunoassay (RIA) procedures as described previously [75] and according to the assay kit manufacturer's instructions.

### Isobaric tag for relative and absolute quantification (iTRAQ)

Proteomic analyses of the hypothalami were conducted using iTRAQ, as described previously [44]. Briefly, iTRAQ chemistry labeling reagents were obtained from Applied Biosystems (Bedford, MA). WT and tgHD rat tissue samples were treated in parallel throughout the labeling procedure. The generic labeling protocol consisted of the following steps: protein reduction and cysteine blocking, digestion of proteins with trypsin, labeling of peptides with iTRAQ reagents, combining the samples that were to be compared, strong cation exchange chromatography, desalting with solid phase extraction, and LC/MS/MS analysis. Briefly, protein sample pellets (generated by trichloroacetic acid precipitation) were dissolved in dissolution buffer (0.5 M triethyammonium bicarbonate, TEAB), to give a 5 µg/µL concentration. Subsequently, 20 µL (100 µg) of each mixture was aliquoted and 1 µL denaturant (2% SDS) was added. Reducing reagent (2 µL, 50 mM TECP) was added and the tubes were incubated at 60°C for 1 hour. The proprietary (methyl methanethiosulphonate, Applied Biosystems, Bedford, MA) cysteine-blocking reagent (1 µL, 200 mM MMTS) was added and incubated for another 10 minutes at room temperature. Trypsin (Promega, Madison, WI) was reconstituted in water; 10 µL of the solution containing 10 µg of trypsin was added and incubated overnight at 37°C. Before labeling, the reagents were dissolved in ethanol and the contents of one vial were transferred to a sample tube. The labeling took place for 1 h at room temperature. The following labels were used for hypothalamus samples: 114-tgHD (C1)-2 pooled Huntington's disease animals; 115-tgHD (C2)-2 pooled Huntington's disease animals; 116-tgHD (C3)-2 pooled Huntington's disease animals; 117- WT(C4)-2 pooled Wild-type animals. After labeling, sample tubes for WT and tgHD for separate tissues were combined and dried down to a volume of 50 µL to reduce the content of ethanol prior to strong cation exchange (SCX) chromatography.

### Mass Spectrometry analysis

An Applied Biosystems QStar mass spectrometer was used for the isolation and collision-induced dissociation of input peptides. The electrospray voltage typically maintained was 2.5 kV. Mass spectrometer calibration was performed with a mixture of CsI (MW132.9049), synthetic peptide ALILTLVS (829.5393) and verapamil (455.2904). The general conditions for mass ion identification and isobaric mass tag resolution were: Scan Events: 1: Survey 400–1200 Da; 2–4: Data dependent MS/MS on 3 most intense ions from 1. For collision energy: rolling collision energy (automatically set according to the m/z of precursor), increased for 20% due to iTRAQ tags. The exclusion time used for analysis was 60 seconds. Mass tolerance was set to 0.15 atomic mass units for precursor and 0.1 atomic mass units for fragmented ions. Data analysis was performed using the ProQuant program suit (Applied Biosystems, Bedford, MA). Protein identification was performed using the most recently updated rodent SwissProt database. Raw peptide identification results were processed to generate a minimal set of proteins, as previously described, using the Paragon Algorithm (Applied Biosystems, Bedford, MA). Briefly, the raw peptide identification results from the Paragon Algorithm searches were further processed by the ProGroup Algorithm (Applied Biosystems, Bedford, MA) within the Protein Pilot software before final display. The ProGroup Algorithm uses the peptide identification results to determine the minimal set of proteins that can be reported for a given protein confidence threshold. For each protein, ProGroup reports two score types for each protein: unused ProtScore and total ProtScore. The total ProtScore is a measurement of all the peptide evidence for a protein (analogous to commonly reported protein scores). The unused ProtScore is a measurement of all the peptide evidence for a protein that is not better explained by a higher-ranking protein. Hence unused ProtScore is calculated by using the unique peptides that are not linked to a higher-ranking protein. The protein confidence threshold cutoff for this study was ProtScore 2.0 (unused) with at least two peptides with a 95% confidence. Proteins identified with mass tag changes (ratio = 1.2 or 0.8) that were consistent between two independent biological experiments were manually validated and quantified by two independent analysts. These arbitrary cutoffs for expression variation have been implemented by multiple researchers [44], [76]. Peak areas for each of the signature ions (114, 115, 116, 117) were obtained and corrected according to the manufacturers' instructions to account for isotopic overlap. Only those signature ions with intensities less than 1500 counts were used for quantification, greater than 1500 counts results in detector saturation. To calculate the relative protein levels, proteins with a statistically significant label ratio of 114/115/116∶117 greater or equal to 1.2 were considered to be proteins elevated tgHD versus WT samples. Proteins with a significant label ratio of 114/115/116∶117 less than or equal to 0.8 were indicative of down-regulated proteins in tgHD versus WT. The relative expression level for ‘up-regulated’ proteins was calculated as follows: the mean of 114/115/116∶117 label ratio. The eventual relative expression level of ‘down-regulated proteins’ was calculated as follows: the mean of 114/115/116∶117 label ratio and then made negative to indicate the relative direction of expression compared to control.

### Western blot analysis

Various proteins identified by iTRAQ were validated using western blot, as described previously [77]. 20 µg samples of the hypothalamus were mixed with a denaturing and reducing Laemmli buffer and resolved by SDS-PAGE. The proteins were then electrotransferred from the gel onto a polyvinylenedifluoride (PVDF: NEN Life Sciences) membrane. The membrane was treated with a Tris-buffered saline solution supplemented with Tween-20, NP-40 and 5% bovine serum albumin to block non-specific antibody interactions. Primary antibodies (1∶500–1000) were applied to the PVDF membrane for 1 hour at room temperature and proteins were identified by the application of an alkaline-phosphatase-conjugated secondary antibody (1∶10000) that recognized the species type of the primary antibody. The PVDF membrane was then exposed to an enzyme-linked chemifluorescent developing substrate (GE Heathcare, Piscataway, NJ) and was scanned using a GE Typhoon 9410 phosphorimager. Western blot images were then quantified using GE ImageQuant 5.1 software. The primary antibodies were obtained from the following sources: Lamin A antibody (sc-20680) and Gspt2 antibody (sc-135899), obtained from Santa Cruz; GFAP (#3670), Pebp1 (#4742), PKM2 (#3198), Flotillin-2 (#3436) and mTOR (#2983) antibodies were from the Cell Signaling Technology (Danvers, MA); beta-actin (A5316) antibody was purchased from Sigma (St. Louis, MO).

### Bioinformatics - Protein pathway analyses and Latent Semantic Indexing

KEGG (Kyoto Encyclopedia of Genes and Genomes: http://www.genome.jp/kegg/) pathway clustering was performed using WebGestalt algorithms (http://bioinfo.vanderbilt.edu/webgestalt), as described previously [78], [79]. We employed basic quantitative parameters for the significant inclusion of KEGG pathway groups, *i.e.* each group needed a minimum population of two proteins from the input experimental set and to possess a probability significance of enrichment compared to a background dataset of less than 0.05 (hypergeometric test of significance). The degree of enrichment (R: expressed as a ratio) of KEGG pathway group was calculated as follows: R = O/E where O is the observed protein number in the KEGG pathway, E is the expected protein number in the KEGG pathway, and a P value is given for the pathways with R>1 to indicate the significance of enrichment. Based on those parameters, a hybrid score is calculated with the formula of R*(−log_10_(P)). GO analysis is an enrichment analysis for the Gene Ontology categories. The result is visualized in a directed acyclic graph (DAG) in order to maintain the relationship among the enriched GO categories. For each GO category, a hybrid score was calculated as well, based upon the same formula as KEGG analysis. The gene symbols of significantly altered hypothalamic proteins identified by iTRAQ were interrogated against a range of keywords using Latent Semantic Indexing (LSI) from GeneIndexer, as described previously [80]–[82]. Briefly, gene symbols of significantly altered proteins were uploaded into the Computable Genomix program (http://www.computablegenomix.com/). GeneIndexer correlates the strength of association, using LSI, between specific genes (proteins) in a dataset with user-defined interrogation terms. GeneIndexer employs a comprehensive murine database of over 2×10^6^ scientific abstracts to perform this text-gene correlation analysis. LSI facilitates the specific textual interrogation of an input dataset with a specific interrogation term, *e.g.* diabetes, to ascertain which of the input dataset genes/proteins are associated with the interrogation term. The possible LSI correlation scores for a gene/protein to be associated with an input interrogation term range from 0 to 1. A score of 0 indicates neutrality of correlation, while scores ranging from 0 to −1 indicate a strengthening lack of correlation of the gene/protein to the input interrogation term. Proteins with a significant correlation (value >0.1) were extracted and labeled as either up-regulated (red) or down-regulated (green).

### Statistical analyses

Both the Student's t-test and 2-way ANOVA analysis were employed in this study. A Student's t-test was used for comparison between tgHD rats and WT rats and the longitudinal alterations, in which 3-month aged animals were regarded as a control in each genotype group. Two-way ANOVA analysis was also performed to evaluate all plasma parameters, where genotype and age were regarded as 2 independent variables. p<0.05 was considered statistically significant throughout the study. Error bars on graphs represent the ±95% confidence interval. All data represent means ± S.E.M. (standard error of mean).

## Supporting Information

Table S1 **Two-way ANOVA analysis of plasma parameters.** All plasma parameters were also analyzed by 2-way ANOVA, in which age and genotype were regarded as 2 independent factors. P value^a^ indicates the significance for the comparison between tgHD rats and the age-matched WT rats. P value^b^ indicates the significance for the comparison between 2 different ages in tgHD rats.
(DOC)

Table S2 **Significantly regulated proteins in HD rat hypothalamic tissue compared to WT animals.** The Mean ratios of significantly altered proteins between Huntington's rats (tgHD) and wild-type control animals (WT) are listed below. The mean ratios > = 1.2 indicate increased expression in tgHD rats compared to WT and mean ratios < = 0.8 indicate reduced expression compared to WT.
(DOC)

Table S3 **GeneIndexer Latent Semantic Indexing (LSI) analysis of significantly up-regulated hypothalamic proteins.** The search terms used were: “Huntingtin,” “chorea,” “diabetes,” and “insulin.” A score of “0” indicates no significant correlation; a score of “1” indicates a significant correlation, p≤0 .05.
(DOC)

Table S4 **GeneIndexer Latent Semantic Indexing (LSI) analysis of significantly down-regulated hypothalamic proteins.** The search terms used were: “Huntingtin,” “chorea,” “diabetes,” and “insulin.” A score of “0” indicates no significant correlation; a score of “1” indicates a significant correlation, p≤0.05.
(DOC)

## References

[1] Landles C, Bates GP (2004) Huntingtin and the molecular pathogenesis of Huntington's disease. Fourth in molecular medicine review series. EMBO Rep 5: 958–963. 15459747 10.1038/sj.embor.7400250 PMC1299150

[2] Li SH, Li XJ (2004) Huntington and its Role in Neuronal Degeneration. Neuroscientist 10: 467–475. 15359012 10.1177/1073858404266777

[3] MacDonald ME, Ambrose CM, Duyao MP, Myers RH, Lin C, et al (1993) A novel gene containing a trinucleotide repeat that is expanded and unstable on Huntington's disease chromosomes. Cell 72: 971–983. 8458085 10.1016/0092-8674(93)90585-e

[4] Langbehn DR, Brinkman RR, Falush D, Paulsen JS, Hayden MR (2004) on behalf of an International Huntington's Disease Collaborative G (2004) A new model for prediction of the age of onset and penetrance for Huntington's disease based on CAG length. Clinical Genetics 65: 267–277. 15025718 10.1111/j.1399-0004.2004.00241.x

[5] Dewhurst K, Oliver J (1970) Huntington's disease of young people. Eur Neurol 3: 278–289. 4245793 10.1159/000113980

[6] Brandt J, Folstein SE, Folstein MF (1988) Differential cognitive impairment in Alzheimer's disease and Huntington's disease. Ann Neurol 23: 555–561. 2970248 10.1002/ana.410230605

[7] Bamford KA, Caine ED, Kido DK, Cox C, Shoulson I (1995) A prospective evaluation of cognitive decline in early Huntington's disease: functional and radiographic correlates. Neurology 45: 1867–1873. 7477984 10.1212/wnl.45.10.1867

[8] Shiwach R (1994) Psychopathology in Huntington's disease patients. Acta Psychiatr Scand 90: 241–246. 7831992 10.1111/j.1600-0447.1994.tb01587.x

[9] Sanberg PR, Fibiger HC, Mark RF (1981) Body weight and dietary factors in Huntington's disease patients compared with matched controls. Med J Aust 1: 407–409. 6454826 10.5694/j.1326-5377.1981.tb135681.x

[10] Djousse L, Knowlton B, Cupples LA, Marder K, Shoulson I, et al (2002) Weight loss in early stage of Huntington's disease. Neurology 59: 1325–1330. 12427878 10.1212/01.wnl.0000031791.10922.cf

[11] Hamilton JM, Wolfson T, Peavy GM, Jacobson MW, Corey-Bloom J (2004) Rate and correlates of weight change in Huntington's disease. Journal of Neurology, Neurosurgery & Psychiatry 75: 209–212. 10.1136/jnnp.2003.017822 PMC1738924 14742590

[12] Ahmad Aziz N, Pijl H, Frölich M, Maurits van der Graaf AW, Roelfsema F, et al (2009) Leptin secretion rate increases with higher CAG repeat number in Huntington's disease patients. Clinical Endocrinology 73: 206–211. 19548952 10.1111/j.1365-2265.2009.03661.x

[13] Podolsky S, Leopold NA (1977) Abnormal glucose tolerance and arginine tolerance tests in Huntington's disease. Gerontology 23: 55–63. 136379 10.1159/000212174

[14] Kremer HP, Roos RA, Frolich M, Radder JK, Nieuwenhuijzen Kruseman AC, et al (1989) Endocrine functions in Huntington's disease. A two-and-a-half years follow-up study. J Neurol Sci 90: 335–344. 2525607 10.1016/0022-510x(89)90120-2

[15] Martin B, Golden E, Carlson OD, Pistell P, Zhou J, et al (2009) Exendin-4 Improves Glycemic Control, Ameliorates Brain and Pancreatic Pathologies, and Extends Survival in a Mouse Model of Huntington's Disease. Diabetes 58: 318–328. 18984744 10.2337/db08-0799 PMC2628604

[16] Trejo A, Tarrats RM, Alonso ME, Boll M-C, Ochoa A, et al (2004) Assessment of the nutrition status of patients with Huntington's disease. Nutrition 20: 192–196. 14962685 10.1016/j.nut.2003.10.007

[17] Morton AJ, Wood NI, Hastings MH, Hurelbrink C, Barker RA, et al (2005) Disintegration of the Sleep-Wake Cycle and Circadian Timing in Huntington's Disease. J Neurosci 25: 157–163. 15634777 10.1523/JNEUROSCI.3842-04.2005 PMC6725210

[18] Hult S, Soylu R, Björklund T, Belgardt BF, Mauer J, et al (2011) Mutant huntingtin causes metabolic imbalance by disruption of hypothalamic neurocircuits. Cell Metabolism 13(4): 428–39. 21459327 10.1016/j.cmet.2011.02.013

[19] Aylward EH, Sparks BF, Field KM, Yallapragada V, Shpritz BD, et al (2004) Onset and rate of striatal atrophy in preclinical Huntington disease. Neurology 63: 66–72. 15249612 10.1212/01.wnl.0000132965.14653.d1

[20] Kipps CM, Duggins AJ, Mahant N, Gomes L, Ashburner J, et al (2005) Progression of structural neuropathology in preclinical Huntington's disease: a tensor based morphometry study. Journal of Neurology, Neurosurgery & Psychiatry 76: 650–655. 10.1136/jnnp.2004.047993 PMC1739615 15834021

[21] Paulsen JS, Magnotta VA, Mikos AE, Paulson HL, Penziner E, et al (2006) Brain structure in preclinical Huntington's Disease. Biological Psychiatry 59: 57–63. 16112655 10.1016/j.biopsych.2005.06.003

[22] Wolf RC, Vasic N, Schönfeldt-Lecuona C, Landwehrmeyer GB, Ecker D (2007) Dorsolateral prefrontal cortex dysfunction in presymptomatic Huntington's disease: evidence from event-related fMRI. Brain 130: 2845–2857. 17855375 10.1093/brain/awm210

[23] Rosas HD, Hevelone ND, Zaleta AK, Greve DN, Salat DH, et al (2005) Regional cortical thinning in preclinical Huntington disease and its relationship to cognition. Neurology 65: 745–747. 16157910 10.1212/01.wnl.0000174432.87383.87

[24] Soneson C, Fontes M, Zhou Y, Denisov V, Paulsen JS, et al (2010) Early changes in the hypothalamic region in prodromal Huntington disease revealed by MRI analysis. Neurobiology of Disease 40: 531–543. 20682340 10.1016/j.nbd.2010.07.013 PMC2955781

[25] Sawiak SJ, Wood NI, Williams GB, Morton AJ, Carpenter TA, et al (2009) Voxel-based morphometry in the R6/2 transgenic mouse reveals differences between genotypes not seen with manual 2D morphometry. Neurobiology of Disease 33: 20–27. 18930824 10.1016/j.nbd.2008.09.016

[26] Björkqvist M, Leavitt BR, Nielsen JE, Landwehrmeyer B, Ecker D, et al (2007) Cocaine- and amphetamine-regulated transcript is increased in Huntington disease. Movement Disorders 22: 1952–1954. 17722045 10.1002/mds.21447

[27] Gabery S, Murphy K, Schultz K, Loy C, McCusker E, et al (2010) Changes in key hypothalamic neuropeptide populations in Huntington disease revealed by neuropathological analyses. Acta Neuropathologica 120: 777–788. 20821223 10.1007/s00401-010-0742-6

[28] Mangiarini L, Sathasivam K, Seller M, Cozens B, Harper A, et al (1996) Exon 1 of the HD gene with an expanded CAG repeat is sufficient to cause a progressive neurological phenotype in transgenic mice. Cell 87: 493–506. 8898202 10.1016/s0092-8674(00)81369-0

[29] McBride JL, Ramaswamy S, Gasmi M, Bartus RT, Herzog CD, et al (2006) Viral delivery of glial cell line-derived neurotrophic factor improves behavior and protects striatal neurons in a mouse model of Huntington's disease. Proceedings of the National Academy of Sciences 103: 9345–9350. 10.1073/pnas.0508875103 PMC1482612 16751280

[30] von Hörsten S, Schmitt I, Nguyen HP, Holzmann C, Schmidt T, et al (2003) Transgenic rat model of Huntington's disease. Human Molecular Genetics 12: 617–624. 12620967 10.1093/hmg/ddg075

[31] Nguyen HP, Kobbe P, Rahne H, Wörpel T, Jäger B, et al (2006) Behavioral abnormalities precede neuropathological markers in rats transgenic for Huntington's disease. Human Molecular Genetics 15: 3177–3194. 16984963 10.1093/hmg/ddl394

[32] Martin B, Golden E, Keselman A, Stone M, Mattson MP, et al (2008) Therapeutic perspectives for the treatment of Huntington's disease: treating the whole body. Histol Histopathol 23: 237–250. 17999380 10.14670/hh-23.237 PMC2657556

[33] van der Burg JMM, Björkqvist M, Brundin P (2009) Beyond the brain: widespread pathology in Huntington's disease. The Lancet Neurology 8: 765–774. 19608102 10.1016/S1474-4422(09)70178-4

[34] Cai H, Cong WN, Ji S, Rothman S, Maudsley S, et al (2012) Metabolic dysfunction in Alzheimer's disease and related neurodegenerative disorders. Curr Alzheimer Res 9: 5–17. 22329649 10.2174/156720512799015064 PMC4097094

[35] Hult S, Schultz K, Soylu R, Petersén A (2010) Hypothalamic and neuroendocrine changes in Huntington's disease. Curr Drug Targets 11: 1237–1249. 20594177 10.2174/1389450111007011237

[36] Lalic NM, Maric J, Svetel M, Jotic A, Stefanova E, et al (2008) Glucose Homeostasis in Huntington disease. Arch neurol 65: 476–480. 18413469 10.1001/archneur.65.4.476

[37] Farrer LA (1985) Diabetes Mellitus in Huntington's disease. Clin Genet 27: 62–67. 3156696 10.1111/j.1399-0004.1985.tb00185.x

[38] Popovic V, Svetel M, Djurovic M, Petrovic S, Doknic M, et al (2004) Circulating and cerebrospinal fluid ghrelin and leptin: potential role in altered body weight in Huntington's disease. Eur J Endocrinol 151: 451–5. 15476444 10.1530/eje.0.1510451

[39] Scherbaum WA (1998) The role of amylin in the physiology of glycemic control. Exp Clin Endocrinol Diabetes 106: 97–102. 9628238 10.1055/s-0029-1211958

[40] Boey D, Sainsbury A, Herzog H (2007) The role of peptide YY in regulating glucose homeostasis. Peptides 28: 390–395. 17210210 10.1016/j.peptides.2006.07.031

[41] Tomita M (2011) Islet amu;oid polypeptide in pancreatic islets from type 1 diabetic subjects. Islets 3: 166–174. 21633185 10.4161/isl.3.4.15875 PMC3154447

[42] Hashimoto K-I, Ito Y, Tanahashi H, Hayashi M, Yamakita N, et al (2012) Hyperglycemic Chorea-Ballism or Acute Exacerbation of Huntington's Chorea? Huntington's DiseaseUnmasked by Diabetic Ketoacidosis in Type 1 Diabetes Mellitus. Clin Endocrinol Metab [Epub ahead of print]. 10.1210/jc.2012-1190 22745234

[43] Hurlbert MS, Zhou W, Wasmeier C, Kaddis FG, Hutton JC, et al (1999) Mice transgenic for an expanded CAG repeat in the Huntington's disease gene develop diabetes. Diabetes 48: 649–651. 10078572 10.2337/diabetes.48.3.649

[44] Martin B, Brenneman R, Becker KG, Gucek M, Cole RN, et al (2008) iTRAQ Analysis of Complex Proteome Alterations in 3×TgAD Alzheimer's Mice: Understanding the Interface between Physiology and Disease. PLoS ONE 3: e2750. 18648646 10.1371/journal.pone.0002750 PMC2453232

[45] Ding WG, Gromada J (1997) Protein kinase A-dependent stimulation of exocytosis in mouse pancreatic beta-cells by glucose-dependent insulinotropic polypeptide. Diabetes 46: 615–621. 9075801 10.2337/diab.46.4.615

[46] Schwartz TW, Holst JJ, Fahrenkrug J, Jensen SL, Nielsen OV, et al (1978) Vagal, cholinergic regulation of pancreatic polypeptide secretion. J Clin Invest 61: 781–789. 641155 10.1172/JCI108992 PMC372593

[47] Baggio LL, Drucker DJ (2007) Biology of Incretins: GLP-1 and GIP. Gastroenterology 132: 2131–2157. 17498508 10.1053/j.gastro.2007.03.054

[48] Field BCT, Chaudhri OB, Bloom SR (2010) Bowels control brain: gut hormones and obesity. Nat Rev Endocrinol 6: 444–453. 20585346 10.1038/nrendo.2010.93

[49] Faivre E, Hamilton A, Holscher C (2012) Effects of acute and chronic administration of GIP analogues on cognition, synaptic plasticity and neurogenesis in mice. European Journal of Pharmacology 674: 294–306. 22115896 10.1016/j.ejphar.2011.11.007

[50] Phan J, Hickey MA, Zhang P, Chesselet MF, Reue K (2009) Adipose tissue dysfunction tracks disease progression in two Huntington's disease mouse models. Hum Mol Genet 18: 1006–1016. 19124532 10.1093/hmg/ddn428 PMC2649017

[51] Leoni V, Mariotti C, Nanetti L, Salvatore E, Squitieri F, et al (2011) Whole body cholesterol metabolism is impaired in Huntington's disease. Neurosci Lett 494: 245–9. 21406216 10.1016/j.neulet.2011.03.025

[52] Valenza M, Leoni V, Karasinska JM, Petricca L, Fan J, et al (2010) Cholesterol defect is marked across multiple rodent models of Huntington's disease and is manifest in astrocytes. J Neurosci 30: 10844–10850. 20702713 10.1523/JNEUROSCI.0917-10.2010 PMC3842469

[53] Salvatore E, Rinaldi C, Tucci T, Di Maio L, Di Somma C, et al (2011) Growth hormone response to arginine test differentiates between two subgroups of Huntington's disease patients. J Neurol Neurosurg Psychiatry 82: 543–7. 20884675 10.1136/jnnp.2010.208553

[54] Whittier JR (1976) Letter: Asphyxiation, bulimia, and insulin levels in Huntington disease (chorea). JAMA 235: 1423–1424. 130504

[55] Peterson A, Hult S, Kirik D (2009) Huntington's Disease :New Perspectives Based on Neuroendocrine Changes in Rodent Models. Neurodegenerative Diseases 6: 154–164. 19521064 10.1159/000225377

[56] Chadwick W, Martin B, Chapter MC, Park SS, Wang L, et al (2012) GIT2 acts as a potential keystone protein in functional hypothalamic networks associated with age-related phenotypic changes in rats. PLoS One 7 5: e36975. 22606319 10.1371/journal.pone.0036975 PMC3351446

[57] Middeldorp J, Hol EM (2011) GFAP in health and disease. Prog Neurobiol 93: 421–43. 21219963 10.1016/j.pneurobio.2011.01.005

[58] De Sandre-Giovannoli A, Chaouch M, Kozlov S, Vallat JM, Tazir M, et al (2002) Homozygous defects in LMNA, encoding lamin A/C nuclear-envelope proteins, cause autosomal recessive axonal neuropathy in human (Charcot-Marie-Tooth disorder type 2) and mouse. Am J Hum Genet 70: 726–36. 11799477 10.1086/339274 PMC384949

[59] Zhao WQ, Lu B (2007) Expression of annexin A2 in GABAergic interneurons in the normal rat brain. J Neurochem 100: 1211–23. 17316400 10.1111/j.1471-4159.2006.04311.x

[60] Lalli G (2009) RalA and the exocyst complex influence neuronal polarity through PAR-3 and aPKC. J Cell Sci 122: 1499–506. 19383721 10.1242/jcs.044339

[61] Lotz GP, Legleiter J, Aron R, Mitchell EJ, Huang SY, et al (2010) Hsp70 and Hsp40 functionally interact with soluble mutant huntingtin oligomers in a classic ATP-dependent reaction cycle. J Biol Chem 285: 38183–93. 20864533 10.1074/jbc.M110.160218 PMC2992252

[62] Braunstein KE, Eschbach J, Ròna-Vörös K, Soylu R, Mikrouli E, et al (2010) A point mutation in the dynein heavy chain gene leads to striatal atrophy and compromises neurite outgrowth of striatal neurons. Hum Mol Genet 19: 4385–98. 20807776 10.1093/hmg/ddq361 PMC3298848

[63] Bellinger FP, Bellinger MT, Seale LA, Takemoto AS, Raman AV, et al (2011) Glutathione peroxidase 4 is associated with neuromelanin in Substantia Nigra and dystrophic axons in putamen of Parkinson's brain. Mol Neurodegener 6: 8. 21255396 10.1186/1750-1326-6-8 PMC3037910

[64] Raza H (2011) Dual localization of glutathione S-transferase in the cytosol and mitochondria: implications in oxidative stress, toxicity and disease. FEBS J 278: 4243–51. 21929724 10.1111/j.1742-4658.2011.08358.x PMC3204177

[65] Stranahan AM, Lee K, Becker KG, Zhang Y, Maudsley S, et al (2010) Hippocampal gene expression patterns underlying the enhancement of memory by running in aged mice. Neurobiology of Aging 31: 1937–1949. 19070401 10.1016/j.neurobiolaging.2008.10.016 PMC2889222

[66] Irimia JM, Meyer CM, Peper CL, Zhai L, Bock CB, et al (2010) Impaired Glucose Tolerance and Predisposition to the Fasted State in Liver Glycogen Synthase Knock-out Mice. Journal of Biological Chemistry 285: 12851–12861. 20178984 10.1074/jbc.M110.106534 PMC2857087

[67] Holness MJ, Bulmer K, Smith ND, Sugden MC (2003) Investigation of potential mechanisms regulating protein expression of hepatic pyruvate dehydrogenase kinase isoforms 2 and 4 by fatty acids and thyroid hormone. Biochem J 369: 687–695. 12435272 10.1042/BJ20021509 PMC1223128

[68] Mayers RM, Leighton B, Kilgour E (2005) PDH kinase inhibitors: a novel therapy for Type II diabetes? Biochem Soc Trans 33: 367–370. 15787608 10.1042/BST0330367

[69] Spoden GA, Rostek U, Lechner S, Mitterberger M, Mazurek S, et al (2009) Pyruvate kinase isoenzyme M2 is a glycolytic sensor differentially regulating cell proliferation, cell size and apoptotic cell death dependent on glucose supply. Experimental Cell Research 315: 2765–2774. 19563799 10.1016/j.yexcr.2009.06.024

[70] Takeuchi K, Reue K (2009) Biochemistry, physiology, and genetics of GPAT, AGPAT, and lipin enzymes in triglyceride synthesis. American Journal of Physiology - Endocrinology And Metabolism 296: E1195–E1209. 19336658 10.1152/ajpendo.90958.2008 PMC2692402

[71] Pohl J, Ring A, Herrmann T, Stremmel W (2004) New concepts of cellular fatty acid uptake: role of fatty acid transport proteins and of caveolae. Proceedings of the Nutrition Society 63: 259–262. 15294040 10.1079/PNS2004341

[72] López M, Vidal-Puig A (2008) Brain lipogenesis and regulation of energy metabolism. Curr Opin Clin Nutr Metab Care 11: 483–490. 18542011 10.1097/MCO.0b013e328302f3d8

[73] Martin B, Shin YK, White CM, Ji S, Kim W, et al (2010) Vasoactive intestinal peptide-null mice demonstrate enhanced sweet taste preference, dysglycemia, and reduced taste bud leptin receptor expression. Diabetes 59: 1143–52. 20150284 10.2337/db09-0807 PMC2857894

[74] Martin B, Pearson M, Kebejian L, Golden E, Keselman A, et al (2007) Sex-dependent metabolic, neuroendocrine, and cognitive responses to dietary energy restriction and excess. Endocrinology 148: 4318–33. 17569758 10.1210/en.2007-0161 PMC2622430

[75] Rothman SM, Herdener N, Camandola S, Texel SJ, Mughal MR, et al (2012) 3×TgAD mice exhibit altered behavior and elevated Aβ after chronic mild social stress. Neurobiol Aging 33: 830.e1–12. 10.1016/j.neurobiolaging.2011.07.005 PMC4074356 21855175

[76] Griffiths SD, Burthem J, Unwin RD, Holyoake TL, Melo JV, et al (2007) The use of isobaric tag peptide labeling (iTRAQ) and mass spectrometry to examine rare, primitive hematopoietic cells from patients with chronic myeloid leukemia. Mol Biotechnol 36: 81–89. 17914187 10.1007/s12033-007-0005-5

[77] Martin B, Brenneman R, Golden E, Walent T, Becker KG (2009) et al. Growth factor signals in neural cells: coherent patterns of interaction control multiple levels of molecular and phenotypic responses. J Biol Chem 284: 2493–511. 19038969 10.1074/jbc.M804545200 PMC2629109

[78] Chadwick W, Boyle JP, Zhou Y, Wang L, Park SS, et al (2011) Multiple oxygen tension environments reveal diverse patterns of transcriptional regulation in primary astrocytes. PLoS One 6: e21638. 21738745 10.1371/journal.pone.0021638 PMC3124552

[79] Martin B, Pearson M, Brenneman R, Golden E, Wood W, et al (2009) Gonadal transcriptome alterations in response to dietary energy intake: sensing the reproductive environment. PLoS One 4: e4146. 19127293 10.1371/journal.pone.0004146 PMC2607546

[80] Chadwick W, Zhou Y, Park SS, Wang L, Mitchell N, et al (2010) Minimal peroxide exposure of neuronal cells induces multifaceted adaptive responses. PLoS One 5: e14352. 21179406 10.1371/journal.pone.0014352 PMC3003681

[81] Chadwick W, Brenneman R, Martin B, Maudsley S (2010) Complex and multidimensional lipid raft alterations in a murine model of Alzheimer's disease. Int J Alzheimers Dis 604792. 10.4061/2010/604792 PMC2997345 21151659

[82] Chadwick W, Park SS, Zhou Y, Wang L, Brenneman R, et al (2011) Repetitive peroxide exposure reveals pleiotropic mitogen-activated protein kinase signaling mechanisms. J Signal Transduct pii 636951. 10.1155/2011/636951 PMC3023409 21258655

