# Correction: Altered Hypothalamic Protein Expression in a Rat Model of Huntington's Disease

**DOI:** 10.1371/annotation/677c26e3-ce52-4837-853a-63c4ed7d72c0

**Published:** 2012-11-15

**Authors:** Wei-na Cong, Huan Cai, Rui Wang, Caitlin M. Daimon, Stuart Maudsley, Kerstin Raber, Fabio Canneva, Stephan von Hörsten, Bronwen Martin

There was an error in the Funding statement.

The statement should read: This work was supported by the Intramural Program of the National Institute on Aging, National Institutes of Health.

