# Supplementary material for: Altered Hypothalamic Protein Expression in a Rat Model of Huntington's Disease
Source: PLoS One. 2012 Oct 18;7(10):e47240. doi: 10.1371/journal.pone.0047240 (PMC3475691; doi:10.1371/journal.pone.0047240)
Supplement: Table S1 — Two-way ANOVA analysis of plasma parameters. All plasma parameters were also analyzed by 2-way ANOVA, in which age and genotype were regarded as 2 independent factors. P valuea indicates the significance for the comparison between tgHD rats and the age-matched WT rats. P valueb indicates the significance for the comparison between 2 different ages in tgHD rats. (DOC) [file pone.0047240.s001.doc]

**Table S1. Two-way ANOVA analysis of plasma parameters.** All plasma parameters were also analyzed by 2-way ANOVA, in which age and genotype were regarded as 2 independent factors. P value^a^ indicates the significance for the comparison between tgHD rats and the age-matched WT rats. P value^b^ indicates the significance for the comparison between 2 different ages in tgHD rats.

| Parameter | group | age | mean | Std. Error | P  value^a^ | *vs.* age (within the same genotype) | P value^b^ |
| --- | --- | --- | --- | --- | --- | --- | --- |
| **Glucose** | tgHD | 3 month | 111.42 | 9.123 | 0.951 | 9 month | 0.249 |
|  |  |  |  |  |  | 12 month | 0.109 |
|  |  | 9 month | 120.99 | 4.883 | 0.524 | 3 month | 0.249 |
|  |  |  |  |  |  | 12 month | 0.009 |
|  |  | 12 month | 97.90 | 3.018 | 0.630 | 3 month | 0.109 |
|  |  |  |  |  |  | 9 month | 0.009 |
| **Insulin** | tgHD | 3 month | 2736.19 | 194.2 | 0.000 | 9 month | 0.762 |
|  |  |  |  |  |  | 12 month | 0.111 |
|  |  | 9 month | 2891.68 | 398.5 | 0.411 | 3 month | 0.762 |
|  |  |  |  |  |  | 12 month | 0.064 |
|  |  | 12 month | 1846.05 | 97.18 | 0.210 | 3 month | 0.111 |
|  |  |  |  |  |  | 9 month | 0.064 |
| **Adiponectin** | tgHD | 3 month | 6131.4 | 412.4 | 0.650 | 9 month | 0.127 |
|  |  |  |  |  |  | 12 month | 0.755 |
|  |  | 9 month | 4948.0 | 266.2 | 0.402 | 3 month | 0.127 |
|  |  |  |  |  |  | 12 month | 0.070 |
|  |  | 12 month | 6368.2 | 486.6 | 0.772 | 3 month | 0.755 |
|  |  |  |  |  |  | 9 month | 0.070 |
| **leptin** | tgHD | 3 month | 6713.7 | 354.7 | 0.214 | 9 month | 0.030 |
|  |  |  |  |  |  | 12 month | 0.541 |
|  |  | 9 month | 9036.3 | 522.0 | 0.593 | 3 month | 0.030 |
|  |  |  |  |  |  | 12 month | 0.098 |
|  |  | 12 month | 7302.2 | 444.4 | 0.036 | 3 month | 0.541 |
|  |  |  |  |  |  | 9 month | 0.098 |
| **Ghrelin** | tgHD | 3 month | 609.0 | 25.5 | 0.828 | 9 month | 0.039 |
|  |  |  |  |  |  | 12 month | 0.005 |
|  |  | 9 month | 769.1 | 54.3 | 0.805 | 3 month | 0.039 |
|  |  |  |  |  |  | 12 month | 0.305 |
|  |  | 12 month | 850.3 | 106.1 | 0.651 | 3 month | 0.005 |
|  |  |  |  |  |  | 9 month | 0.305 |
| **Amylin** | tgHD | 3 month | 93.0 | 3.27 | 0.000 | 9 month | 0.466 |
|  |  |  |  |  |  | 12 month | 0.002 |
|  |  | 9 month | 86.8 | 10.68 | 0.862 | 3 month | 0.466 |
|  |  |  |  |  |  | 12 month | 0.012 |
|  |  | 12 month | 62.1 | 5.71 | 0.525 | 3 month | 0.002 |
|  |  |  |  |  |  | 9 month | 0.012 |
| **GIP** | tgHD | 3 month | 214.7 | 9.4 | 0.158 | 9 month | 0.623 |
|  |  |  |  |  |  | 12 month | 0.038 |
|  |  | 9 month | 198.6 | 15.1 | 0.355 | 3 month | 0.623 |
|  |  |  |  |  |  | 12 month | 0.102 |
|  |  | 12 month | 143.7 | 14.4 | 0.206 | 3 month | 0.038 |
|  |  |  |  |  |  | 9 month | 0.102 |
| **PP** | tgHD | 3 month | 38.4 | 4.9 | 0.812 | 9 month | 0.011 |
|  |  |  |  |  |  | 12 month | 0.274 |
|  |  | 9 month | 66.5 | 8.7 | 0.708 | 3 month | 0.011 |
|  |  |  |  |  |  | 12 month | 0.129 |
|  |  | 12 month | 53.5 | 6.5 | 0.052 | 3 month | 0.274 |
|  |  |  |  |  |  | 9 month | 0.129 |
| **PYY** | tgHD | 3 month | 78.4 | 2.9 | 0.003 | 9 month | 0.531 |
|  |  |  |  |  |  | 12 month | 0.518 |
|  |  | 9 month | 83.9 | 5.2 | 0.718 | 3 month | 0.531 |
|  |  |  |  |  |  | 12 month | 0.222 |
|  |  | 12 month | 72.4 | 6.3 | 0.232 | 3 month | 0.518 |
|  |  |  |  |  |  | 9 month | 0.222 |
| **Corticosterone** | tgHD | 3 month | 111.8 | 33.9 | 0.566 | 9 month | 0.208 |
|  |  |  |  |  |  | 12 month | 0.521 |
|  |  | 9 month | 184.1 | 34.7 | 0.909 | 3 month | 0.208 |
|  |  |  |  |  |  | 12 month | 0.503 |
|  |  | 12 month | 146.1 | 26.8 | 0.069 | 3 month | 0.521 |
|  |  |  |  |  |  | 9 month | 0.503 |
| **TG** | tgHD | 3 month | 55.2 | 4.89 | 0.000 | 9 month | 0.360 |
|  |  |  |  |  |  | 12 month | 0.981 |
|  |  | 9 month | 65.4 | 8.83 | 0.709 | 3 month | 0.360 |
|  |  |  |  |  |  | 12 month | 0.349 |
|  |  | 12 month | 55.0 | 5.78 | 0.288 | 3 month | 0.981 |
|  |  |  |  |  |  | 9 month | 0.349 |
| **TC** | tgHD | 3 month | 64.0 | 1.73 | 0.137 | 9 month | 0.001 |
|  |  |  |  |  |  | 12 month | 0.010 |
|  |  | 9 month | 85.6 | 3.45 | 0.180 | 3 month | 0.001 |
|  |  |  |  |  |  | 12 month | 0.289 |
|  |  | 12 month | 79.6 | 6.03 | 0.334 | 3 month | 0.010 |
|  |  |  |  |  |  | 9 month | 0.289 |
| **HDL** | tgHD | 3 month | 14.6 | 0.49 | 0.052 | 9 month | 0.950 |
|  |  |  |  |  |  | 12 month | 0.081 |
|  |  | 9 month | 14.5 | 1.40 | 0.782  . | 3 month | 0.950 |
|  |  |  |  |  |  | 12 month | 0.092 |
|  |  | 12 month | 12.0 | 0.57 | 0.908 | 3 month | 0.081 |
|  |  |  |  |  |  | 9 month | 0.092 |
| **LDL** | tgHD | 3 month | 43.8 | 0.88 | 0.379 | 9 month | 0.000 |
|  |  |  |  |  |  | 12 month | 0.009 |
|  |  | 9 month | 57.4 | 2.91 | 0.337 | 3 month | 0.000 |
|  |  |  |  |  |  | 12 month | 0.214 |
|  |  | 12 month | 53.2 | 3.17 | 0.165 | 3 month | 0.009 |
|  |  |  |  |  |  | 9 month | 0.214 |
| **HDL/LDL** | tgHD | 3 month | 0.333 | 0.01 | 0.165 | 9 month | 0.012 |
|  |  |  |  |  |  | 12 month | 0.002 |
|  |  | 9 month | 0.252 | 0.02 | 0.695 | 3 month | 0.012 |
|  |  |  |  |  |  | 12 month | 0.507 |
|  |  | 12 month | 0.232 | 0.02 | 0.393 | 3 month | 0.002 |
|  |  |  |  |  |  | 9 month | 0.507 |
