# Supplementary material for: Altered Hypothalamic Protein Expression in a Rat Model of Huntington's Disease
Source: PLoS One. 2012 Oct 18;7(10):e47240. doi: 10.1371/journal.pone.0047240 (PMC3475691; doi:10.1371/journal.pone.0047240)
Supplement: Table S2 — Significantly regulated proteins in HD rat hypothalamic tissue compared to WT animals. The Mean ratios of significantly altered proteins between Huntington's rats (tgHD) and wild-type control animals (WT) are listed below. The mean ratios > = 1.2 indicate increased expression in tgHD rats compared to WT and mean ratios < = 0.8 indicate reduced expression compared to WT. (DOC) [file pone.0047240.s002.doc]

**Table S2. Significantly regulated proteins in HD rat hypothalamic tissue compared to WT animals.** The mean ratios of significantly altered proteins between Huntington’s rats (tgHD) and wild-type control animals (WT) are listed below. The mean ratios >=1.2 indicate increased expression in tgHD rats compared to WT and mean ratios <=0.8 indicate reduced expression compared to WT.

| **Protein Description** | **Gene symbol** | **Mean ratio (114, 115, 116)** |
| --- | --- | --- |
| tubulin alpha-1A chain [Mus musculus] | Tuba1a | 2.145 |
| COMM domain-containing protein 5 [Mus musculus] | Commd5 | 2.114 |
| short coiled-coil protein isoform b [Mus musculus] | Scoc | 1.815 |
| glutathione S-transferase Mu 7 [Mus musculus] | Gstm7 | 1.773 |
| phospholipid hydroperoxide glutathione peroxidase, nuclear isoform 2 precursor [Mus musculus] | Gpx4 | 1.762 |
| PREDICTED: similar to C50H11.1 [Rattus norvegicus] | Acsf2 | 1.608 |
| G1 to S phase transition 2 [Rattus norvegicus] | Gspt2 | 1.602 |
| hypothetical protein LOC217310 [Mus musculus] | Dym | 1.581 |
| ubiquitin specific protease 32 [Rattus norvegicus] | Usp32 | 1.498 |
| sphingosine kinase 2 [Rattus norvegicus] | Sphk2 | 1.463 |
| bridging integrator 1 [Rattus norvegicus] | Bin1 | 1.446 |
| haloacid dehalogenase-like hydrolase domain containing 2 [Rattus norvegicus] | Hdhd2 | 1.445 |
| [Pyruvate dehydrogenase [lipoamide]] kinase isozyme 2, mitochondrial precursor [Rattus norvegicus] | Pdk2 | 1.436 |
| serine/threonine-protein kinase 25 [Mus musculus] | Stk25 | 1.416 |
| pyruvate kinase, muscle [Rattus norvegicus] | Pkm2 | 1.414 |
| glycogen [starch] synthase, muscle [Mus musculus] | Gys1 | 1.396 |
| ras-related protein Ral-A precursor [Mus musculus] | Rala | 1.393 |
| COP9 signalosome complex subunit 7a isoform 1 [Mus musculus] | Cops7a | 1.380 |
| BTB (POZ) domain containing 17 [Rattus norvegicus] | Btbd17 | 1.376 |
| PREDICTED: similar to Ca2+-dependent activator for secretion protein 2 [Rattus norvegicus] |  | 1.366 |
| PREDICTED: hypothetical protein [Rattus norvegicus] | Glt1d1 | 1.366 |
| rho GTPase-activating protein RICH2 isoform 2 [Mus musculus] | AU040829 | 1.360 |
| ubiquitin specific peptidase 10 [Rattus norvegicus] | Usp10 | 1.350 |
| PI-PLC X domain-containing protein 3 [Mus musculus] | Plcxd3 | 1.342 |
| H2A histone family, member X [Rattus norvegicus] | H2afx | 1.341 |
| calcineurin-like phosphoesterase domain containing 1 [Rattus norvegicus] | Cpped1 | 1.341 |
| N-terminal kinase-like protein [Mus musculus] | Scyl1 | 1.340 |
| myosin light chain 6B [Mus musculus] | Myl6b | 1.335 |
| pre-mRNA-processing factor 19 [Mus musculus] | Prpf19 | 1.333 |
| TBC1 domain family, member 24 [Rattus norvegicus] | Tbc1d24 | 1.325 |
| eukaryotic translation initiation factor 1 [Mus musculus] | Eif1 | 1.324 |
| GATS-like protein 1 [Mus musculus] | Gatsl1 | 1.319 |
| myeloid leukemia factor 2 homolog [Mus musculus] | Mlf2 | 1.319 |
| phosphatidylinositol-4-phosphate 5-kinase type-1 gamma isoform 2 [Mus musculus] | Pip5k1c | 1.318 |
| 1,4-alpha-glucan-branching enzyme [Mus musculus] | Gbe1 | 1.308 |
| amyloid beta A4 precursor protein-binding family A member 2 [Mus musculus] | Apba2 | 1.304 |
| tubulin beta-2A chain [Mus musculus] | Tubb2a | 1.299 |
| hypothetical protein LOC296565 [Rattus norvegicus] |  | 1.288 |
| serine/threonine-protein kinase 24 [Mus musculus] | Stk24 | 1.285 |
| E3 ubiquitin-protein ligase RNF123 [Mus musculus] | rnf123 | 1.284 |
| sorting nexin family member 30 [Rattus norvegicus] | Snx30 | 1.284 |
| PREDICTED: similar to hect (homologous to the E6-AP (UBE3A) carboxyl terminus) domain and RCC1 (CHC1)-like domain (RLD) 2 [Rattus norvegicus] |  | 1.283 |
| nuclear receptor coactivator 5 [Rattus norvegicus] | Ncoa5 | 1.283 |
| long-chain-fatty-acid--CoA ligase 3 isoform b [Mus musculus] | Acsl3 | 1.282 |
| hyaluronan binding protein 4 [Rattus norvegicus] | Habp4 | 1.279 |
| dynamin 1 [Rattus norvegicus] | Dnm1 | 1.273 |
| suppressor of Ty 5 homolog [Mus musculus] | Supt5h | 1.270 |
| cysteine desulfurase, mitochondrial [Rattus norvegicus] | Nfs1 | 1.269 |
| PREDICTED: similar to haloacid dehalogenase-like hydrolase domain containing 3 [Rattus norvegicus] | LOC682946 | 1.267 |
| shroom family member 2 [Rattus norvegicus] | Shroom2 | 1.265 |
| 1-acylglycerol-3-phosphate O-acyltransferase 1 [Rattus norvegicus] | Agpat1 | 1.260 |
| phosphofurin acidic cluster sorting protein 1 [Rattus norvegicus] | Pacs1 | 1.255 |
| tubulin beta-2B chain [Mus musculus] | Tubb2b | 1.253 |
| phosphatidylethanolamine binding protein 1 [Rattus norvegicus] | Pebp1 | 1.249 |
| catechol-O-methyltransferase domain containing 1 [Rattus norvegicus] | Comtd1 | 1.249 |
| PREDICTED: similar to phosphoribosylformylglycinamidine synthase [Rattus norvegicus] | Pfas | 1.244 |
| Rho GTPase activating protein 5 [Rattus norvegicus] | Arhgap5 | 1.244 |
| IQ motif and Sec7 domain 3 [Rattus norvegicus] | Iqsec3 | 1.240 |
| F-box and leucine-rich repeat protein 16 [Rattus norvegicus] | Fbxl16 | 1.238 |
| choline/ethanolamine kinase [Rattus norvegicus] | Chka | 1.233 |
| thioredoxin-related transmembrane protein 4 precursor [Mus musculus] | Tmx4 | 1.232 |
| arfaptin-2 [Mus musculus] | Arfip2 | 1.231 |
| disco-interacting protein 2 homolog B isoform 2 [Mus musculus] | Dip2b | 1.230 |
| SH3-domain GRB2-like 3 [Rattus norvegicus] | Sh3gl3 | 1.226 |
| tubulin beta-3 chain [Mus musculus] | Tubb3 | 1.224 |
| tubulin beta-2C chain [Mus musculus] | Tubb2c | 1.221 |
| SH2B adapter protein 1 isoform 2 [Rattus norvegicus] | Sh2bpsm1 | 1.220 |
| TAO kinase 1 [Rattus norvegicus] | Taok1 | 1.219 |
| PREDICTED: similar to small nuclear ribonucleoparticle-associated protein [Rattus norvegicus] | RGD1563375_predicted | 1.217 |
| SEC14-like protein 2 [Rattus norvegicus] | Sec14l2 | 1.216 |
| PC4 and SFRS1-interacting protein [Mus musculus] | Psip1 | 1.212 |
| dynamin 3 [Rattus norvegicus] | Dnm3 | 1.211 |
| PREDICTED: similar to sarcolemma associated protein [Rattus norvegicus] | Ccdc136 | 1.207 |
| serine/threonine-protein kinase mTOR [Rattus norvegicus] | Mtor | 1.206 |
| glutaryl-Coenzyme A dehydrogenase [Rattus norvegicus] | Gcdh | 1.206 |
| PREDICTED: similar to sacsin [Rattus norvegicus] | Sacs | 1.205 |
| G protein-coupled receptor associated sorting protein 1 [Rattus norvegicus] | Gprasp1 | 1.204 |
| glycerophosphodiester phosphodiesterase domain-containing protein 1 [Mus musculus] | Gdpd1 | 1.203 |
| centaurin, alpha 1 [Rattus norvegicus] | Adap1 | 1.203 |
| PREDICTED: similar to Protein CXorf17 homolog [Rattus norvegicus] |  | 1.201 |
| neurofibromin [Mus musculus] | Nf1 | 1.201 |
| phospholipase D family, member 3 [Rattus norvegicus] | Pld3 | 1.200 |
| cytidine monophospho-N-acetylneuraminic acid synthetase [Rattus norvegicus] | Cmas | 1.200 |
| versican core protein isoform 3 [Mus musculus] | Vcan | 1.200 |
| guanine nucleotide binding protein, alpha q polypeptide [Rattus norvegicus] | Gnaq | 0.800 |
| interferon-induced GTP-binding protein Mx2 [Rattus norvegicus] | Mx2 | 0.800 |
| nucleolar and coiled-body phosphoprotein 1 [Rattus norvegicus] | Nolc1 | 0.800 |
| nucleophosmin [Mus musculus] | Npm1 | 0.799 |
| lamina-associated polypeptide 2, isoforms alpha/zeta isoform zeta [Mus musculus] | Tmpo | 0.799 |
| PREDICTED: hypothetical protein [Mus musculus] |  | 0.799 |
| sodium- and chloride-dependent GABA transporter 3 [Rattus norvegicus] | Slc6a11 | 0.799 |
| parathymosin [Mus musculus] | Ptms | 0.799 |
| protein ALEX isoform g [Mus musculus] | Gnas | 0.799 |
| cystatin-B [Rattus norvegicus] | Cstb | 0.798 |
| heat shock 70kD protein 1B [Rattus norvegicus] | Hspa1b | 0.797 |
| sideroflexin 3 [Rattus norvegicus] | Sfxn3 | 0.797 |
| NADH dehydrogenase (ubiquinone) Fe-S protein 3 [Rattus norvegicus] | Ndufs3 | 0.796 |
| NADH dehydrogenase ubiquinone flavoprotein 2 precursor [Rattus norvegicus] | Ndufv2 | 0.796 |
| guanine nucleotide-binding protein G(i) subunit alpha-2 [Rattus norvegicus] | Gnai2 | 0.796 |
| hydroxyacyl-Coenzyme A dehydrogenase precursor [Rattus norvegicus] | Hadh | 0.796 |
| serine (or cysteine) peptidase inhibitor, clade B, member 9 [Rattus norvegicus] | Serpinb9 | 0.796 |
| NADH dehydrogenase [ubiquinone] 1 alpha subcomplex subunit 9, mitochondrial [Rattus norvegicus] | Ndufa9 | 0.795 |
| Bcl2-associated athanogene 3 [Rattus norvegicus] | Bag3 | 0.795 |
| carboxymethylenebutenolidase homolog [Rattus norvegicus] | Cmbl | 0.795 |
| glutathione S-transferase alpha 4 [Rattus norvegicus] | Gsta4 | 0.794 |
| NADPH--cytochrome P450 reductase [Mus musculus] | Por | 0.794 |
| serine (or cysteine) proteinase inhibitor, clade B (ovalbumin), member 6 [Rattus norvegicus] | Serpinb6a | 0.794 |
| protein tweety homolog 1 [Rattus norvegicus] | Ttyh1 | 0.794 |
| cleavage and polyadenylation specific factor 7, 59kDa [Rattus norvegicus] | Cpsf7 | 0.793 |
| PREDICTED: similar to NADH dehydrogenase (ubiquinone) Fe-S protein 6 [Mus musculus] |  | 0.792 |
| RNA-binding protein 8A isoform b [Mus musculus] | Rbm8a | 0.792 |
| cysteine-rich protein 2 [Rattus norvegicus] | Csrp2 | 0.792 |
| PREDICTED: similar to 3-ketoacyl-CoA thiolase B, peroxisomal precursor (Beta-ketothiolase B) (Acetyl-CoA acyltransferase B) (Peroxisomal 3-oxoacyl-CoA thiolase B) [Rattus norvegicus] |  | 0.792 |
| transmembrane emp24 domain-containing protein 10 precursor [Mus musculus] | Tmed10 | 0.791 |
| fermitin family homolog 2 [Rattus norvegicus] | Fermt2 | 0.790 |
| TAP-binding protein [Rattus norvegicus] | Tapbp | 0.789 |
| cAMP-dependent protein kinase type I-alpha regulatory subunit [Mus musculus] | Prkar1a | 0.787 |
| ATP synthase, H+ transporting, mitochondrial F0 complex, subunit c, isoform 1 [Mus musculus] | Atp5g1 | 0.787 |
| spectrin beta chain, brain 1 isoform 1 [Mus musculus] | Spnb2 | 0.786 |
| plexin B2 [Rattus norvegicus] | Plxnb2 | 0.786 |
| adenylate kinase 3 [Rattus norvegicus] | Ak3 | 0.785 |
| nucleobindin 2 precursor [Rattus norvegicus] | Nucb2 | 0.785 |
| indolethylamine N-methyltransferase [Mus musculus] | Inmt | 0.784 |
| PREDICTED: similar to NADH-ubiquinone oxidoreductase PDSW subunit (Complex I-PDSW) (CI-PDSW) isoform 1 [Rattus norvegicus] |  | 0.784 |
| lymphocyte cytosolic protein 1 [Rattus norvegicus] | Lcp1 | 0.784 |
| myosin, heavy chain 9, non-muscle [Rattus norvegicus] | Myh9 | 0.784 |
| endonuclease domain-containing 1 protein precursor [Mus musculus] | Endod1 | 0.784 |
| glial fibrillary acidic protein [Rattus norvegicus] | Gfap | 0.784 |
| all-trans-13,14-dihydroretinol saturase precursor [Rattus norvegicus] | Retsat | 0.783 |
| phosphoglucomutase-2 [Mus musculus] | Pgm1 | 0.783 |
| mitochondrial trifunctional protein, beta subunit precursor [Rattus norvegicus] | Hadhb | 0.782 |
| peptidylprolyl isomerase B [Rattus norvegicus] | Ppib | 0.782 |
| PREDICTED: similar to Filamin-C (Gamma-filamin) (Filamin-2) (Protein FLNc) (Actin-binding-like protein) (ABP-L) (ABP-280-like protein) isoform 3 [Rattus norvegicus] |  | 0.781 |
| delta-1-pyrroline-5-carboxylate dehydrogenase, mitochondrial [Rattus norvegicus] | Aldh4a1 | 0.780 |
| propionyl Coenzyme A carboxylase, beta polypeptide precursor [Rattus norvegicus] | Pccb | 0.778 |
| flotillin-2 isoform 2 [Mus musculus] | Flot2 | 0.776 |
| prohibitin-2 [Mus musculus] | Phb2 | 0.775 |
| armadillo repeat protein deleted in velo-cardio-facial syndrome homolog [Mus musculus] | Arvcf | 0.775 |
| liver glycogen phosphorylase [Rattus norvegicus] | Pygl | 0.775 |
| solute carrier family 2, facilitated glucose transporter member 1 [Mus musculus] | Slc2a1 | 0.775 |
| protein disulfide-isomerase A4 precursor [Rattus norvegicus] | Pdia4 | 0.774 |
| splicing factor 1 isoform 2 [Mus musculus] | Sf1 | 0.773 |
| SH3 domain-binding glutamic acid-rich-like protein [Mus musculus] | Sh3bgrl | 0.773 |
| protein tyrosine phosphatase, receptor type, A precursor [Rattus norvegicus] | Ptpra | 0.773 |
| B-cell receptor-associated protein 31 [Rattus norvegicus] | Bcap31 | 0.773 |
| cytochrome c oxidase subunit II [Jaculus jaculus] | COX2 | 0.771 |
| radixin [Rattus norvegicus] | Rdx | 0.771 |
| 4F2 cell-surface antigen heavy chain [Rattus norvegicus] | Slc3a2 | 0.771 |
| potassium voltage-gated channel subfamily D member 2 [Mus musculus] | Kcnd2 | 0.770 |
| NADH dehydrogenase [ubiquinone] 1 alpha subcomplex subunit 5 [Rattus norvegicus] | Ndufa5 | 0.769 |
| prolyl 4-hydroxylase, beta polypeptide [Rattus norvegicus] | P4hb | 0.768 |
| thioredoxin domain-containing protein 5 precursor [Mus musculus] | Txndc5 | 0.768 |
| mitochondrial trifunctional protein, alpha subunit precursor [Rattus norvegicus] | Hadha | 0.765 |
| catenin delta-1 isoform 3 [Mus musculus] | Ctnnd1 | 0.765 |
| surfeit locus protein 4 [Mus musculus] | Surf4 | 0.764 |
| sodium/calcium exchanger 1 isoform B [Mus musculus] | Slc8a1 | 0.764 |
| rho guanine nucleotide exchange factor 2 [Rattus norvegicus] | Arhgef2 | 0.762 |
| propionyl-coenzyme A carboxylase, alpha polypeptide [Rattus norvegicus] | Pcca | 0.762 |
| CDGSH iron sulfur domain-containing protein 1 [Mus musculus] | Cisd1 | 0.761 |
| PREDICTED: similar to AFG3-like protein 2 (Paraplegin-like protein) [Rattus norvegicus] |  | 0.761 |
| lamin-B1 [Mus musculus] | Lmnb1 | 0.759 |
| ribophorin II [Rattus norvegicus] | Rpn2 | 0.759 |
| voltage-dependent anion channel 2 [Rattus norvegicus] | Vdac2 | 0.759 |
| ADP/ATP translocase 1 [Rattus norvegicus] | Slc25a4 | 0.758 |
| voltage-dependent anion-selective channel protein 1 [Mus musculus] | Vdac1 | 0.757 |
| PREDICTED: hypothetical protein [Mus musculus] |  | 0.757 |
| PREDICTED: similar to Dehydrogenase/reductase SDR family member 7 precursor (Retinal short-chain dehydrogenase/reductase 4) [Rattus norvegicus] |  | 0.756 |
| cadherin 2 precursor [Rattus norvegicus] | Cdh2 | 0.756 |
| mitochondrial carrier homolog 1 [Mus musculus] | Mtch1 | 0.756 |
| cytochrome c oxidase subunit IV isoform 1 precursor [Rattus norvegicus] | Cox4i1 | 0.756 |
| PREDICTED: similar to desmuslin isoform M isoform 2 [Rattus norvegicus] |  | 0.755 |
| spartin [Rattus norvegicus] | Spg20 | 0.754 |
| PDZ and LIM domain 5 [Rattus norvegicus] | Pdlim5 | 0.754 |
| hepatocyte cell adhesion molecule precursor [Mus musculus] | Hepacam | 0.753 |
| ubiquinol-cytochrome c reductase core protein I precursor [Rattus norvegicus] | Uqcrc1 | 0.753 |
| eukaryotic translation initiation factor 3 subunit M [Mus musculus] | Eif3m | 0.753 |
| diacylglycerol kinase theta [Mus musculus] | Dgkq | 0.753 |
| NADH dehydrogenase (ubiquinone) 1 beta subcomplex, 9 [Rattus norvegicus] | Ndufb9 | 0.752 |
| bisphosphoglycerate mutase [Rattus norvegicus] | Bpgm | 0.752 |
| guanine nucleotide-binding protein G(I)/G(S)/G(T) subunit beta-1 [Mus musculus] | Gnb1 | 0.750 |
| signal recognition particle receptor subunit alpha [Mus musculus] | Srpr | 0.749 |
| polypyrimidine tract-binding protein 1 isoform 2 [Mus musculus] | Ptbp1 | 0.747 |
| excitatory amino acid transporter 2 isoform a [Rattus norvegicus] | Slc1a2 | 0.747 |
| pleiotrophin [Mus musculus] | Ptn | 0.746 |
| cold-inducible RNA-binding protein [Mus musculus] | Cirbp | 0.745 |
| trypsin 10 [Mus musculus] | try10 | 0.745 |
| sorcin [Rattus norvegicus] | Sri | 0.744 |
| ezrin [Rattus norvegicus] | Ezr | 0.744 |
| neuronal membrane glycoprotein M6-b [Mus musculus] | Gpm6b | 0.743 |
| ATP synthase subunit d, mitochondrial [Rattus norvegicus] | Atp5h | 0.742 |
| filamin, beta [Rattus norvegicus] | Flnb | 0.741 |
| aldehyde dehydrogenase 1A1 [Rattus norvegicus] | Aldh1a1 | 0.740 |
| protein disulfide-isomerase A3 precursor [Mus musculus] | Pdia3 | 0.738 |
| dodecenoyl-Coenzyme A delta isomerase precursor [Rattus norvegicus] | Dci | 0.737 |
| 6-phosphogluconate dehydrogenase, decarboxylating [Mus musculus] | Pgd | 0.736 |
| ATP synthase subunit b, mitochondrial precursor [Rattus norvegicus] | Atp5f1 | 0.736 |
| annexin A6 [Rattus norvegicus] | Anxa6 | 0.734 |
| calponin 3, acidic [Rattus norvegicus] | Cnn3 | 0.733 |
| CD166 antigen precursor [Rattus norvegicus] | Alcam | 0.732 |
| alcohol dehydrogenase 1 [Rattus norvegicus] | Adh1 | 0.730 |
| phosphomannomutase 2 [Mus musculus] | Pmm2 | 0.729 |
| lamin A isoform C2 [Rattus norvegicus] | Lmna | 0.727 |
| luc7-like protein 3 [Mus musculus] | Luc7l3 | 0.726 |
| actinin, alpha 1 [Rattus norvegicus] | Actn1 | 0.724 |
| complement C4 precursor [Rattus norvegicus] | C4b | 0.724 |
| protein phosphatase 1, regulatory (inhibitor) subunit 1A [Rattus norvegicus] | Ppp1r1a | 0.724 |
| PREDICTED: similar to valosin isoform 1 [Mus musculus] |  | 0.723 |
| PREDICTED: similar to destrin [Rattus norvegicus] |  | 0.722 |
| NAD(P) transhydrogenase, mitochondrial precursor [Mus musculus] | Nnt | 0.719 |
| guanine nucleotide-binding protein G(I)/G(S)/G(T) subunit beta-2 [Mus musculus] | Gnb2 | 0.719 |
| PREDICTED: similar to formin 3 CG33556-PA [Rattus norvegicus] |  | 0.718 |
| PREDICTED: similar to nuclear mitotic apparatus protein 1 isoform 1 [Rattus norvegicus] |  | 0.717 |
| glycine dehydrogenase (decarboxylating) [Rattus norvegicus] | Gldc | 0.716 |
| nucleoredoxin [Rattus norvegicus] | Nxn | 0.716 |
| acyl-CoA thioesterase 2 [Rattus norvegicus] | Acot2 | 0.714 |
| acetyl-Coenzyme A acyltransferase 2 [Rattus norvegicus] | Acaa2 | 0.714 |
| macrophage-capping protein [Rattus norvegicus] | Capg | 0.713 |
| plasma glutamate carboxypeptidase precursor [Rattus norvegicus] | Pgcp | 0.713 |
| neurofascin isoform 1 precursor [Rattus norvegicus] | Nfasc | 0.713 |
| lipase A precursor [Rattus norvegicus] | Lipa | 0.713 |
| prostaglandin reductase 1 [Rattus norvegicus] | Ptgr1 | 0.713 |
| neurochondrin [Mus musculus] | Ncdn | 0.713 |
| PREDICTED: similar to Adenylosuccinate synthetase isozyme 1 (Adenylosuccinate synthetase, muscle isozyme) (IMP--aspartate ligase 1) (AdSS 1) (AMPSase 1) isoform 1 [Rattus norvegicus] |  | 0.711 |
| PREDICTED: similar to Pre-B-cell leukemia transcription factor interacting protein 1 [Mus musculus] |  | 0.711 |
| PREDICTED: similar to ribosome binding protein 1 isoform 1 [Rattus norvegicus] |  | 0.709 |
| chloride intracellular channel protein 4 [Mus musculus] | Clic4 | 0.709 |
| annexin A11 [Rattus norvegicus] | Anxa11 | 0.709 |
| monocarboxylate transporter 1 [Mus musculus] | Slc16a1 | 0.709 |
| selenoprotein O [Rattus norvegicus] | Selo | 0.707 |
| acyl-CoA synthetase short-chain family member 3, mitochondrial isoform 1 [Mus musculus] | Acss3 | 0.707 |
| voltage-dependent anion-selective channel protein 3 [Mus musculus] | Vdac3 | 0.706 |
| NADH dehydrogenase [ubiquinone] 1 alpha subcomplex subunit 8 [Mus musculus] | Ndufa8 | 0.706 |
| chloride intracellular channel protein 1 [Rattus norvegicus] | Clic1 | 0.703 |
| phosphatidic acid phosphatase type 2B [Rattus norvegicus] | Ppap2b | 0.702 |
| coatomer subunit epsilon [Mus musculus] | Cope | 0.700 |
| alpha-actinin-4 [Rattus norvegicus] | Actn4 | 0.699 |
| ATP synthase, H+ transporting, mitochondrial F1 complex, delta subunit precursor [Rattus norvegicus] | Atp5d | 0.699 |
| serine carboxypeptidase 1 precursor [Rattus norvegicus] | Scpep1 | 0.698 |
| cell division cycle protein 123 homolog [Rattus norvegicus] | Cdc123 | 0.697 |
| phosphate carrier protein, mitochondrial precursor [Rattus norvegicus] | Slc25a3 | 0.696 |
| cytoplasmic dynein 1 heavy chain 1 [Mus musculus] | Dync1h1 | 0.694 |
| ADP/ATP translocase 2 [Mus musculus] | Slc25a5 | 0.694 |
| moesin [Rattus norvegicus] | Msn | 0.691 |
| protein phosphatase 1 regulatory subunit 1B [Rattus norvegicus] | Ppp1r1b | 0.690 |
| mitochondrial inner membrane protein [Mus musculus] | Immt | 0.690 |
| acyl carrier protein, mitochondrial precursor [Mus musculus] | Ndufab1 | 0.682 |
| monoamine oxidase B [Rattus norvegicus] | Maobf3 | 0.681 |
| immunity-related GTPase family, M [Rattus norvegicus] | Irgm | 0.680 |
| phospholemman isoform b precursor [Mus musculus] | Fxyd1 | 0.680 |
| myosin regulatory light chain 12B [Mus musculus] | Mrlc2 | 0.676 |
| PREDICTED: similar to Lymphocyte antigen 6H precursor (Ly-6H) [Mus musculus] |  | 0.675 |
| prothymosin alpha [Rattus norvegicus] | Ptma | 0.675 |
| transmembrane emp24 domain-containing protein 4 precursor [Mus musculus] | Tmed4 | 0.675 |
| nuclease-sensitive element-binding protein 1 [Mus musculus] | Ybx1 | 0.674 |
| nucleoside diphosphate kinase A [Rattus norvegicus] | Nme1 | 0.672 |
| retinol-binding protein 1 [Rattus norvegicus] | Rbp1 | 0.670 |
| PREDICTED: similar to beta-parvin isoform 2 [Mus musculus] |  | 0.669 |
| integrin-linked kinase [Rattus norvegicus] | Ilk | 0.659 |
| calcium modulating ligand [Rattus norvegicus] | Camlg | 0.654 |
| beta-2-microglobulin precursor [Rattus norvegicus] | B2m | 0.653 |
| four and a half LIM domains protein 1 isoform 3 [Mus musculus] | Fhl1 | 0.653 |
| catenin alpha-1 [Mus musculus] | Ctnna1 | 0.653 |
| aldehyde dehydrogenase, mitochondrial precursor [Rattus norvegicus] | Aldh2 | 0.650 |
| spectrin, alpha, erythrocytic 1 [Rattus norvegicus] | Spta1 | 0.650 |
| inter-alpha-inhibitor H4 heavy chain [Rattus norvegicus] | Itih4 | 0.648 |
| PREDICTED: similar to niban protein [Rattus norvegicus] |  | 0.647 |
| multidrug resistance protein 1 [Rattus norvegicus] | Abcb1b | 0.641 |
| regulator of microtubule dynamics protein 3 [Mus musculus] | Fam82a2 | 0.637 |
| catalase [Rattus norvegicus] | Cat | 0.636 |
| extended synaptotagmin-like protein 1 [Rattus norvegicus] | Esyt1 | 0.634 |
| CD81 antigen [Mus musculus] | Cd81 | 0.633 |
| adenosine deaminase [Rattus norvegicus] | Ada | 0.633 |
| selenium binding protein 1 [Rattus norvegicus] | Selenbp1 | 0.630 |
| lamin-A/C isoform C [Mus musculus] | Lmna | 0.630 |
| rho GDP-dissociation inhibitor 2 [Mus musculus] | Arhgdib | 0.630 |
| PREDICTED: similar to Sulfide:quinone oxidoreductase, mitochondrial precursor [Rattus norvegicus] |  | 0.629 |
| calumenin isoform a [Rattus norvegicus] | Calu | 0.628 |
| guanylate binding protein 2 [Rattus norvegicus] | Gbp2 | 0.626 |
| MOCO sulphurase C-terminal domain containing 2 precursor [Rattus norvegicus] | Mosc2 | 0.625 |
| ATP-binding cassette, sub-family G (WHITE), member 3-like 1 [Rattus norvegicus] | Abcg3l1 | 0.623 |
| epoxide hydrolase 1 isoform 2 [Rattus norvegicus] | Ephx1 | 0.620 |
| spectrin beta chain, erythrocyte [Mus musculus] | Spnb1 | 0.620 |
| major vault protein [Rattus norvegicus] | Mvp | 0.620 |
| legumain precursor [Mus musculus] | Lgmn | 0.619 |
| PREDICTED: similar to protein phosphatase 1, regulatory subunit 12C [Rattus norvegicus] |  | 0.613 |
| ATPase, Na+/K+ transporting, beta 3 polypeptide [Rattus norvegicus] | Atp1b3 | 0.610 |
| CD9 antigen [Rattus norvegicus] | Cd9 | 0.609 |
| gelsolin precursor [Rattus norvegicus] | Gsn | 0.608 |
| serpin H1 precursor [Rattus norvegicus] | Serpinh1 | 0.603 |
| transformation related protein 53 inducible protein 11 [Mus musculus] | Trp53i11 | 0.599 |
| phosphoglycerate kinase 1 [Rattus norvegicus] | Pgk1 | 0.599 |
| 5'-nucleotidase, cytosolic III [Rattus norvegicus] | Nt5c3 | 0.596 |
| protein phosphatase 1 regulatory subunit 12A [Rattus norvegicus] | Ppp1r12a | 0.594 |
| PREDICTED: similar to Talin-1 [Rattus norvegicus] |  | 0.591 |
| paraoxonase 1 [Rattus norvegicus] | Pon1 | 0.590 |
| tropomyosin 4 [Rattus norvegicus] | Tpm4 | 0.590 |
| aquaporin-4 isoform 2 [Rattus norvegicus] | Aqp4 | 0.587 |
| cytochrome b5 type A (microsomal) [Rattus norvegicus] | Cyb5a | 0.586 |
| alpha-2-antiplasmin precursor [Rattus norvegicus] | Serpinf2 | 0.582 |
| C-reactive protein precursor [Rattus norvegicus] | Crp | 0.580 |
| transglutaminase 2 [Rattus norvegicus] | Tgm2 | 0.578 |
| fibrinogen gamma chain [Rattus norvegicus] | Fgg | 0.577 |
| carbonic anhydrase 3 [Rattus norvegicus] | Car3 | 0.572 |
| cystine/glutamate transporter [Mus musculus] | Slc7a11 | 0.570 |
| fetuin B precursor [Rattus norvegicus] | Fetub | 0.569 |
| integrin beta-1 precursor [Mus musculus] | Itgb1 | 0.568 |
| sulfotransferase 1A1 [Rattus norvegicus] | Sult1a1 | 0.566 |
| cysteine and glycine-rich protein 1 [Rattus norvegicus] | Csrp1 | 0.565 |
| glutathione peroxidase 1 [Rattus norvegicus] | Gpx1 | 0.562 |
| serine protease inhibitor A3N [Rattus norvegicus] | Serpina3n | 0.561 |
| polymerase I and transcript release factor [Rattus norvegicus] | Ptrf | 0.557 |
| Cd44 molecule [Rattus norvegicus] | Cd44 | 0.556 |
| ras suppressor protein 1 [Mus musculus] | Rsu1 | 0.552 |
| annexin A4 [Rattus norvegicus] | Anxa4 | 0.549 |
| tropomodulin-3 [Mus musculus] | Tmod3 | 0.548 |
| hypothetical protein LOC315963 [Rattus norvegicus] |  | 0.547 |
| alpha-1-antiproteinase precursor [Rattus norvegicus] | Serpina1 | 0.540 |
| Serine protease inhibitor [Rattus norvegicus] | LOC299282 | 0.530 |
| IQ motif containing GTPase activating protein 1 [Rattus norvegicus] | Iqgap1 | 0.528 |
| hemopexin precursor [Rattus norvegicus] | Hpx | 0.526 |
| LIM domain containing preferred translocation partner in lipoma [Rattus norvegicus] | Lpp | 0.516 |
| PREDICTED: similar to promyelocytic leukemia isoform 2 [Rattus norvegicus] |  | 0.515 |
| group specific component precursor [Rattus norvegicus] | Gc | 0.515 |
| complement component 9 [Rattus norvegicus] | C9 | 0.510 |
| serine protease inhibitor A3K [Rattus norvegicus] | Serpina3k | 0.504 |
| tubulin, alpha 1C [Rattus norvegicus] | Tuba1c | 0.500 |
| annexin A5 [Rattus norvegicus] | Anxa5 | 0.500 |
| heat shock protein 1 [Rattus norvegicus] | Hspb1 | 0.500 |
| histidine-rich glycoprotein [Rattus norvegicus] | Hrg | 0.489 |
| PREDICTED: hypothetical protein [Rattus norvegicus] |  | 0.486 |
| CD99 antigen [Rattus norvegicus] | Cd99 | 0.484 |
| apolipoprotein B precursor [Rattus norvegicus] | Apob | 0.479 |
| globin, alpha [Rattus norvegicus] | GloA | 0.476 |
| vinculin [Rattus norvegicus] | Vcl | 0.472 |
| fibrinogen beta chain precursor [Rattus norvegicus] | Fgb | 0.468 |
| annexin A1 [Rattus norvegicus] | Anxa1 | 0.462 |
| transferrin precursor [Rattus norvegicus] | Tf | 0.459 |
| plasminogen [Rattus norvegicus] | Plg | 0.457 |
| complement factor H [Rattus norvegicus] | Cfh | 0.456 |
| albumin precursor [Rattus norvegicus] | Alb | 0.455 |
| pregnancy-zone protein precursor [Rattus norvegicus] | Pzp | 0.452 |
| collagen alpha-1(VI) chain precursor [Mus musculus] | Col9a1 | 0.452 |
| hemoglobin, beta [Rattus norvegicus] | Hbb | 0.447 |
| afamin precursor [Rattus norvegicus] | Afm | 0.446 |
| fibrinogen alpha chain isoform 2 [Rattus norvegicus] | Fga | 0.445 |
| carbonic anhydrase I [Rattus norvegicus] | Car1 | 0.444 |
| serotransferrin precursor [Mus musculus] | Trf | 0.444 |
| galectin-1 [Rattus norvegicus] | Lgals1 | 0.442 |
| orosomucoid 1 precursor [Rattus norvegicus] | Orm1 | 0.441 |
| complement component 3 [Rattus norvegicus] | C3 | 0.437 |
| haptoglobin precursor [Rattus norvegicus] | Hp | 0.434 |
| prostaglandin D2 synthase, brain [Rattus norvegicus] | Ptgds | 0.429 |
| hemoglobin alpha 2 chain [Rattus norvegicus] | LOC360504 | 0.428 |
| transthyretin precursor [Mus musculus] | Ttr | 0.427 |
| PREDICTED: similar to Complement C5 precursor (Hemolytic complement) [Rattus norvegicus] |  | 0.424 |
| hemoglobin alpha, adult chain 2 [Rattus norvegicus] | Hba-a2 | 0.423 |
| PREDICTED: similar to MYL6 protein [Mus musculus] |  | 0.420 |
| aldehyde dehydrogenase 1A2 [Rattus norvegicus] | Aldh1a2 | 0.418 |
| olfactory receptor 24 [Mus musculus] | Olfr24 | 0.417 |
| transgelin 2 [Rattus norvegicus] | Tagln2 | 0.412 |
| beta-glo [Rattus norvegicus] | MGC72973 | 0.403 |
| apolipoprotein A-I precursor [Rattus norvegicus] | Apoa1 | 0.388 |
| vimentin [Rattus norvegicus] | Vim | 0.383 |
| alpha-2-HS-glycoprotein precursor [Rattus norvegicus] | Ahsg | 0.374 |
| ceruloplasmin precursor [Rattus norvegicus] | Cp | 0.368 |
| aminopeptidase O [Mus musculus] | 2010111I01Rik | 0.365 |
| procollagen, type VI, alpha 3 [Rattus norvegicus] | Col6a3 | 0.364 |
| glutathione S-transferase Mu 2 [Rattus norvegicus] | Gstm2 | 0.362 |
| lumican precursor [Rattus norvegicus] | Lum | 0.344 |
| caldesmon 1 [Rattus norvegicus] | Cald1 | 0.344 |
| AHNAK nucleoprotein isoform 1 [Mus musculus] | Ahnak | 0.343 |
| proline/arginine-rich end leucine-rich repeat protein precursor [Rattus norvegicus] | Prelp | 0.343 |
| PREDICTED: similar to Hemoglobin beta-2 subunit (Hemoglobin beta-2 chain) (Beta-2-globin) (Hemoglobin beta chain, minor-form) [Rattus norvegicus] |  | 0.334 |
| branched chain aminotransferase 2, mitochondrial precursor [Rattus norvegicus] | Bcat2 | 0.321 |
| Arg/Abl-interacting protein ArgBP2 [Rattus norvegicus] | Argbp2 | 0.317 |
| annexin A2 [Rattus norvegicus] | Anxa2 | 0.309 |
| band 3 anion transport protein [Rattus norvegicus] | Slc4a1 | 0.302 |
| osteoglycin precursor [Mus musculus] | og | 0.297 |
| PREDICTED: similar to beta-globin isoform 2 [Mus musculus] |  | 0.287 |
| PREDICTED: similar to myosin, heavy polypeptide 11, smooth muscle isoform 1 [Rattus norvegicus] |  | 0.275 |
| transgelin [Rattus norvegicus] | Tagln | 0.270 |
| PREDICTED: similar to esterase 2 [Rattus norvegicus] |  | 0.258 |
| myosin light chain kinase, smooth muscle [Mus musculus] | Mylk | 0.256 |
| filamin, alpha [Rattus norvegicus] | Flna | 0.251 |
| collagen alpha-1(I) chain precursor [Rattus norvegicus] | Col1a1 | 0.250 |
| calponin 1, basic, smooth muscle [Rattus norvegicus] | Cnn1 | 0.238 |
| myosin regulatory light polypeptide 9 [Mus musculus] | Myl9 | 0.234 |
| cochlin precursor [Mus musculus] | Coch | 0.208 |
| protein S100-A9 [Rattus norvegicus] | S100A9 | 0.163 |
| actin, alpha cardiac muscle 1 [Mus musculus] | Actc1 | 0.155 |
| muscle creatine kinase [Rattus norvegicus] | Ckm | 0.151 |
| tropomyosin 1 alpha chain isoform d [Rattus norvegicus] | Tpm1 | 0.140 |
| myelin protein zero precursor [Rattus norvegicus] | Mpz | 0.138 |
| myoglobin [Rattus norvegicus] | Mb | 0.130 |
| tropomyosin 2, beta [Rattus norvegicus] | Tpm2 | 0.124 |
