# Supplementary material for: Altered Hypothalamic Protein Expression in a Rat Model of Huntington's Disease
Source: PLoS One. 2012 Oct 18;7(10):e47240. doi: 10.1371/journal.pone.0047240 (PMC3475691; doi:10.1371/journal.pone.0047240)
Supplement: Table S3 — GeneIndexer Latent Semantic Indexing (LSI) analysis of significantly up-regulated hypothalamic proteins. The search terms used were: “Huntingtin,” “chorea,” “diabetes,” and “insulin.” A score of “0” indicates no significant correlation; a score of “1” indicates a significant correlation, p≤0 .05. (DOC) [file pone.0047240.s003.doc]

**Table S3.** **GeneIndexer Latent Semantic Indexing (LSI) analysis of significantly up-regulated hypothalamic proteins.** The search terms used were: “Huntingtin,” “chorea,” “diabetes,” and “insulin.” A score of “0” indicates no significant correlation; a score of “1” indicates a significant correlation, p ≤ 0.05.

| **Up-regulated Protein Description** | **Gene symbol** | **Huntingtin** | **Chorea** | **Diabetes** | **Insulin** |
| --- | --- | --- | --- | --- | --- |
| 1-acylglycerol-3-phosphate O-acyltransferase 1 [Rattus norvegicus] | agpat1 | 0 | 0 | 1 | 1 |
| glycogen [starch] synthase, muscle [Mus musculus] | gys1 | 0 | 0 | 1 | 1 |
| [Pyruvate dehydrogenase [lipoamide]] kinase isozyme 2, mitochondrial precursor [Rattus norvegicus] | pdk2 | 0 | 0 | 1 | 1 |
| pyruvate kinase, muscle [Rattus norvegicus] | pkm2 | 0 | 0 | 1 | 1 |
| arfaptin-2 [Mus musculus] | arfip2 | 1 | 1 | 0 | 0 |
| PREDICTED: similar to sacsin [Rattus norvegicus] | sacs | 1 | 1 | 0 | 0 |
| SH3-domain GRB2-like 3 [Rattus norvegicus] | sh3gl3 | 1 | 1 | 0 | 0 |
| bridging integrator 1 [Rattus norvegicus] | bin1 | 1 | 0 | 0 | 0 |
| dynamin 1 [Rattus norvegicus] | dnm1 | 1 | 0 | 0 | 0 |
| tubulin beta-2A chain [Mus musculus] | tubb2a | 1 | 0 | 0 | 0 |
| PREDICTED: similar to C50H11.1 [Rattus norvegicus] | acsf2 | 0 | 0 | 0 | 1 |
| Rho GTPase activating protein 5 [Rattus norvegicus] | arhgap5 | 0 | 0 | 0 | 1 |
| ras-related protein Ral-A precursor [Mus musculus] | rala | 0 | 0 | 0 | 1 |
| disco-interacting protein 2 homolog B isoform 2 [Mus musculus] | dip2b | 0 | 1 | 0 | 0 |
