# Supplementary material for: Altered Hypothalamic Protein Expression in a Rat Model of Huntington's Disease
Source: PLoS One. 2012 Oct 18;7(10):e47240. doi: 10.1371/journal.pone.0047240 (PMC3475691; doi:10.1371/journal.pone.0047240)
Supplement: Table S4 — GeneIndexer Latent Semantic Indexing (LSI) analysis of significantly down-regulated hypothalamic proteins. The search terms used were: “Huntingtin,” “chorea,” “diabetes,” and “insulin.” A score of “0” indicates no significant correlation; a score of “1” indicates a significant correlation, p≤0.05. (DOC) [file pone.0047240.s004.doc]

**Table S4.** **GeneIndexer Latent Semantic Indexing (LSI) analysis of significantly down-regulated hypothalamic proteins.** The search terms used were: “Huntingtin,” “chorea,” “diabetes,” and “insulin.” A score of “0” indicates no significant correlation; a score of “1” indicates a significant correlation, p ≤ .05.

| **Down-regulated Protein Description** | **Gene symbol** | **Huntingtin** | **Chorea** | **Diabetes** | **Insulin** |
| --- | --- | --- | --- | --- | --- |
| alpha-2-HS-glycoprotein precursor [Rattus norvegicus] | ahsg | 0 | 1 | 1 | 0 |
| cell division cycle protein 123 homolog [Rattus norvegicus] | cdc123 | 0 | 1 | 1 | 0 |
| CDGSH iron sulfur domain-containing protein 1 [Mus musculus] | cisd1 | 0 | 1 | 1 | 0 |
| fetuin B precursor [Rattus norvegicus] | fetub | 0 | 1 | 1 | 0 |
| hydroxyacyl-Coenzyme A dehydrogenase precursor [Rattus norvegicus] | hadh | 0 | 1 | 1 | 0 |
| MOCO sulphurase C-terminal domain containing 2 precursor [Rattus norvegicus] | mosc2 | 0 | 1 | 1 | 0 |
| NAD(P) transhydrogenase, mitochondrial precursor [Mus musculus] | nnt | 0 | 1 | 1 | 0 |
| nucleoredoxin [Rattus norvegicus] | nxn | 0 | 1 | 1 | 0 |
| propionyl-coenzyme A carboxylase, alpha polypeptide [Rattus norvegicus] | pcca | 0 | 1 | 1 | 0 |
| 6-phosphogluconate dehydrogenase, decarboxylating [Mus musculus] | pgd | 0 | 1 | 1 | 0 |
| polymerase I and transcript release factor [Rattus norvegicus] | ptrf | 0 | 1 | 1 | 0 |
| liver glycogen phosphorylase [Rattus norvegicus] | pygl | 0 | 1 | 1 | 0 |
| all-trans-13,14-dihydroretinol saturase precursor [Rattus norvegicus] | retsat | 0 | 1 | 1 | 0 |
| sideroflexin 3 [Rattus norvegicus] | sfxn3 | 0 | 1 | 1 | 0 |
| solute carrier family 2, facilitated glucose transporter member 1 [Mus musculus] | slc2a1 | 0 | 1 | 1 | 0 |
| thioredoxin domain-containing protein 5 precursor [Mus musculus] | txndc5 | 0 | 1 | 1 | 0 |
| cytoplasmic dynein 1 heavy chain 1 [Mus musculus] | dync1h1 | 1 | 0 | 0 | 0 |
| heat shock 70kD protein 1B [Rattus norvegicus] | hspa1b | 1 | 0 | 0 | 0 |
| lymphocyte cytosolic protein 1 [Rattus norvegicus] | lcp1 | 1 | 0 | 0 | 0 |
| transglutaminase 2 [Rattus norvegicus] | tgm2 | 1 | 0 | 0 | 0 |
| catalase [Rattus norvegicus] | cat | 0 | 1 | 0 | 0 |
| glutathione peroxidase 1 [Rattus norvegicus] | gpx1 | 0 | 1 | 0 | 0 |
| hemoglobin alpha, adult chain 2 [Rattus norvegicus] | hba-a2 | 0 | 1 | 0 | 0 |
| haptoglobin precursor [Rattus norvegicus] | hp | 0 | 1 | 0 | 0 |
| lamin A isoform C2 [Rattus norvegicus] | lmna | 0 | 1 | 0 | 0 |
| orosomucoid 1 precursor [Rattus norvegicus] | orm1 | 0 | 1 | 0 | 0 |
| paraoxonase 1 [Rattus norvegicus] | pon1 | 0 | 1 | 0 | 0 |
| signal recognition particle receptor subunit alpha [Mus musculus] | srpr | 0 | 1 | 0 | 0 |
| branched chain aminotransferase 2, mitochondrial precursor [Rattus norvegicus] | bcat2 | 0 | 0 | 1 | 0 |
| carbonic anhydrase 3 [Rattus norvegicus] | car3 | 0 | 0 | 1 | 0 |
| group specific component precursor [Rattus norvegicus] | gc | 0 | 0 | 1 | 0 |
| guanine nucleotide-binding protein G(i) subunit alpha-2 [Rattus norvegicus] | gnai2 | 0 | 0 | 1 | 0 |
| protein ALEX isoform g [Mus musculus] | gnas | 0 | 0 | 1 | 0 |
| mitochondrial trifunctional protein, alpha subunit precursor [Rattus norvegicus] | hadha | 0 | 0 | 1 | 0 |
| nucleobindin 2 precursor [Rattus norvegicus] | nucb2 | 0 | 0 | 1 | 0 |
| protein phosphatase 1, regulatory (inhibitor) subunit 1A [Rattus norvegicus] | ppp1r1a | 0 | 0 | 1 | 0 |
| protein tyrosine phosphatase, receptor type, A precursor [Rattus norvegicus] | ptpra | 0 | 0 | 1 | 0 |
| monocarboxylate transporter 1 [Mus musculus] | slc16a1 | 0 | 0 | 1 | 0 |
